# Quantification of Factor H Mediated Self vs. Non-self Discrimination by Mathematical Modeling

**DOI:** 10.3389/fimmu.2020.01911

**Published:** 2020-09-02

**Authors:** Alexander Tille, Teresa Lehnert, Peter F. Zipfel, Marc Thilo Figge

**Affiliations:** ^1^Applied Systems Biology, Leibniz Institute for Natural Product Research and Infection Biology—Hans Knöll Institute, Jena, Germany; ^2^Faculty of Biological Sciences, Institute of Microbiology, Friedrich-Schiller-University Jena, Jena, Germany; ^3^Infection Biology, Leibniz Institute for Natural Product Research and Infection Biology—Hans Knöll Institute, Jena, Germany

**Keywords:** complement system, complement regulator factor H, non-self recognition, immune evasion, self-tolerance, mathematical modeling, hybrid differential equation approach

## Abstract

The complement system is part of the innate immune system and plays an important role in the host defense against infectious pathogens. One of the main effects is the opsonization of foreign invaders and subsequent uptake by phagocytosis. Due to the continuous default basal level of active complement molecules, a tight regulation is required to protect the body's own cells (self cells) from opsonization and from complement damage. A major complement regulator is Factor H, which is recruited from the fluid phase and attaches to cell surfaces where it effectively controls complement activation. Besides self cells, pathogens also have the ability to bind Factor H; they can thus escape opsonization and phagocytosis causing severe infections. In order to advance our understanding of the opsonization process at a quantitative level, we developed a mathematical model for the dynamics of the complement system—termed *DynaCoSys model*—that is based on ordinary differential equations for cell surface-bound molecules and on partial differential equations for concentration profiles of the fluid phase molecules in the environment of cells. This hybrid differential equation approach allows to model the complement cascade focusing on the role of active C3b in the fluid phase and on the cell surface as well as on its inactivation on the cell surface. The *DynaCoSys model* enables us to quantitatively predict the conditions under which Factor H mediated complement evasion occurs. Furthermore, investigating the quantitative impact of model parameters by a sensitivity analysis, we identify the driving processes of complement activation and regulation in both the self and non-self regime. The two regimes are defined by a critical Factor H concentration on the cell surface and we use the model to investigate the differential impact of complement model parameters on this threshold value. The dynamic modeling on the surface of pathogens are further relevant to understand pathophysiological situations where Factor H mutants and defective Factor H binding to target surfaces results in pathophysiology such as renal and retinal disease. In the future, this DynaCoSys model will be extended to also enable evaluating treatment strategies of complement-related diseases.

## Introduction

The complement system plays a key role in defending the host against invading pathogens (1–5). Its main task is the recognition, subsequent opsonization and lysis of invading microbes, foreign particles, or altered self cells (6, 7). The central molecule of the complement system is the protein C3b, which binds to cell surfaces, opsonizes the surface and allows subsequent phagocytosis or induction of the lytic terminal complement pathway. C3b results from the cleavage of the intact molecule C3 via three distinct pathways. The classical pathway and lectin pathway are activated, respectively, in the presence of antigen-antibody immune complexes and microbial carbohydrate patterns (8, 9) whereas, the alternative pathway is activated by a spontaneous hydrolysis of C3. Due to this spontaneous emergence of active molecules there is always a basal level of active complement molecules present in blood circulation. Activation and recognition by the complement systems is a double-edged sword. On the one hand, it has to be very sensitive in order to find as many infectious microbes or foreign cells as possible, so that even small fluctuations from the self-state should result into an immediate and corrective response. On the other hand, a tight regulation is required to avoid any unwanted activation and damage to the host. Balance in the host can be disturbed in various ways, such as overshooting complement activation in the context of infectious agents or diseases like sepsis (5, 10), reduced host defense against complement activation caused by genetic defects or autoantibodies (11, 12) or host cells that induce complement activation (e.g., ischemia/reperfusion, burns, apoptotic/necrotic cells). Besides diseases causing complement dysfunction, pathogenic microbes are able to evade the complement system by recruiting complement regulators like Factor H to their surface (13–18). This leads to alterations in the immune response against the pathogen and thus can be associated with serious infections.

For a better understanding of the system's balance, mathematical models can make important contributions to the quantitative understanding of the complex dynamics of the complement system. To study the process of recognizing self vs. non-self cells we here apply a systems biology approach by formulating a mathematical model that is compared to published experimental data. We refer to our newly established model, which describes the dynamics of the complement systems by a set of differential equations, as the *DynaCoSys model*. A main challenge of any quantitative complement model is the estimation of the model parameters. Quantitative time resolved data, especially of surface bound molecules are rare, which in general makes the identification of large mechanistic models difficult. Thus we focus on the main component C3b and introduce effective rates that represent the dynamics of the intermediate products contributing to the formation and decay of C3b. We are able to quantify the complements molecule concentrations on the cell surface and in the surrounding fluid in steady state as well as for the full systems dynamics. Most importantly, in order to arrive at quantitative predictions, we screen model parameters for their differential impact on the critical Factor H concentration that distinguishes between the self and non-self regimes. Our quantitative results are discussed in the light of literature knowledge and in view of applying the model to monitoring treatment of complement-associated diseases.

## Results

### Complement Dynamics of C3b-Opsonization Requires Hybrid Differential Equation Approach

Mathematical models that aim to represent the complement system as a whole by a system of coupled ordinary differential equations (ODEs) are faced with its high complexity and the tremendous number of unknown parameter values (19–21). In addition, ODEs do not account for spatial inhomogeneities in the dynamics and interaction of complement molecules. The spatial distributions of complement molecules are induced by the local accumulation of surface-bound molecules on the cell and decrease in solution as a function of the distance from the cell. This requires a representation by partial differential equations (PDEs) (22, 23); however, modeling complement dynamics by a system of coupled PDEs instead of ODEs would increase the number of unknown parameter values even further. The *DynaCoSys model* is based on a hybrid differential equation (hDE) approach that combines ODEs and PDEs and deliberately reduces the complexity of the system by focusing on the most important complement molecules and by considering spatial inhomogeneities only where necessary. A detailed overview of the *DynaCoSys model* is given in the Supplementary Material Section 1, while in what follows we will only focus on the most relevant aspects.

**Figure 1:**
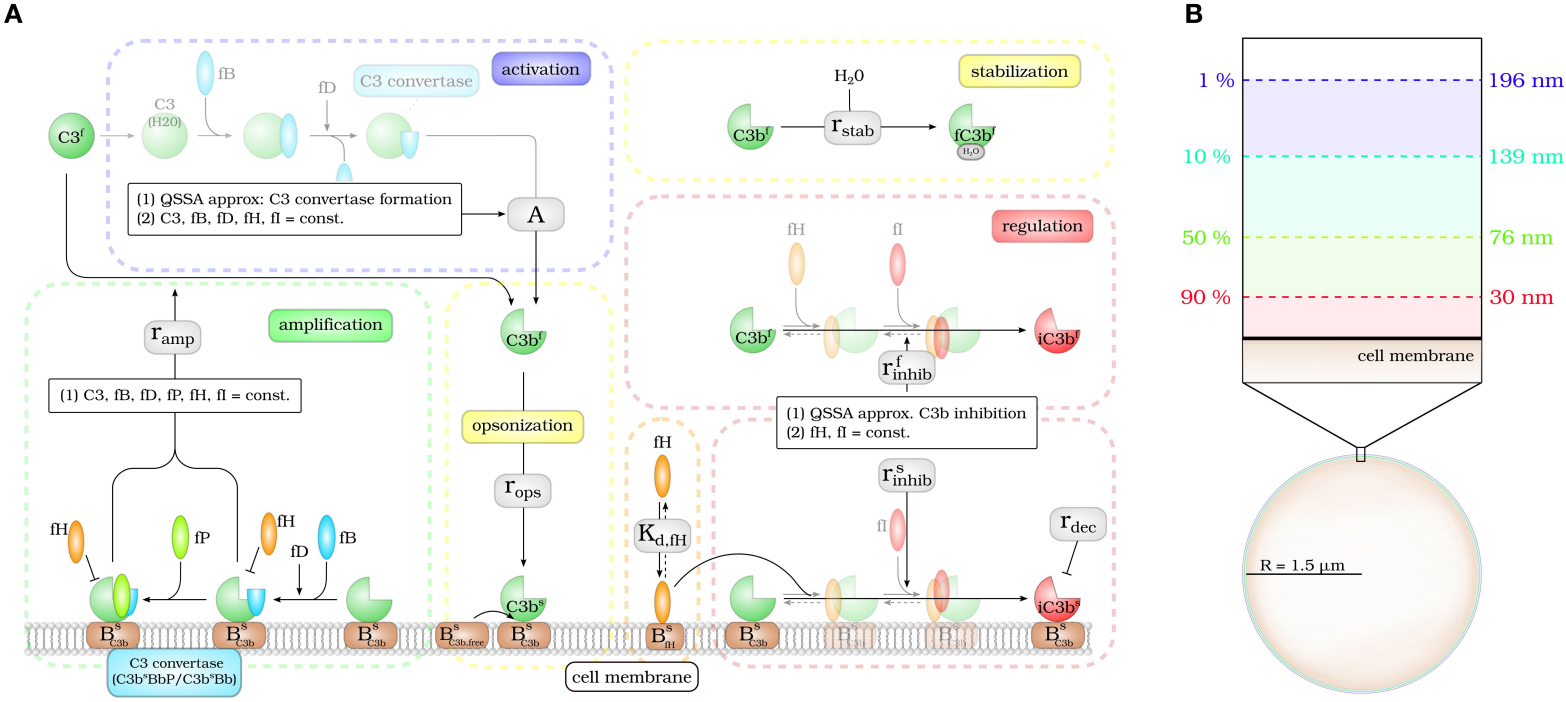
Hybrid differential equation model of the complement system. Complement activation can be divided into five parts: (i) activation, (ii) opsonization, (iii) stabilization, (iv) amplification, and (v) regulation. **(A)** The model focuses on the dynamics of the central component *C3b*: Active *C3b* in the fluid phase, *C3b*^*f*^, results from cleavage of precursor molecule *C3*^*f*^. The interaction of the fluid phase molecule *C3b*^*f*^ with the cell surface is modeled by the interaction with free surface binding sites $B_{C3b,free}^{s}$ and binding sites $B_{C3b}^{s}$ that are occupied with molecules *C3b*^*s*^ on the surface. *C3b*^*f*^ that does not bind to the cell surface gets inactivated via a Factor H mediated inhibition process, or gets stabilized by water molecules and is no longer able to bind to the cell surface. Surface-bound *C3b*^*s*^ can form C3-convertase molecules—*C3b*^*s*^*Bb* and *C3b*^*s*^*BbP*–that cleave *C3*^*f*^ molecules to *C3b*^*f*^ molecules in the vicinity of the cell surface. *C3b*^*s*^ can be inactivated via an inhibition process that is mediated by surface-bound Factor H, whose concentration depends on the concentration of binding sites on the cell surface $B_{fH,max}^{s}$. **(B)** The lifetime of active *C3b*^*f*^ is short such that, depending on the distance from the cell surface, the fraction of molecules that reach the cell surface is small; for example, only 1% at a distance of 196 nm within a simple decay model.

The *DynaCoSys model* focuses on the derivatives of the central complement molecule C3 as the most important molecules (see Figure 1A): in the fluid phase, C3 occurs with concentration *C*3^*f*^ and becomes cleaved into *C*3*a*^*f*^ and *C*3*b*^*f*^. The latter molecule has a highly reactive thioester that enables the molecule to bind covalently to any surface (24). We denote the concentration of surface-bound molecules by *C*3*b*^*s*^; these molecules opsonize cells and allow phagocytosis, but may become inactivated and are then contributing to the concentration *iC*3*b*^*s*^. *C*3*b*^*s*^ can form complexes with other complement components, like Factor B and thereby generate an active enzyme, C3-convertase. This enzyme is able to cleave *C*3^*f*^ and to newly generate *C*3*b*^*f*^ molecules driving a massive amplification loop (2). The C3-convertase complex of the alternative pathway dominates the amplification of *C*3*b*^*s*^ and is responsible for 80–90 % of the total complement activation, even if the complement reaction was initially triggered by the classical or lectin pathway (8). This opsonization process is strictly controlled by regulators (25) usually ensuring that only non-self cells become opsonized (3, 9). The regulators are either present in the fluid phase, attached to cell surfaces or integrated into cell membranes (3). They control the fluid phase activation, the amplification on the surface and they mediate the decay of surface bound *C*3*b*^*s*^ molecules. The soluble regulator Factor H plays a major role in protecting also cells that do not have membrane-bound inhibitors (26). It mediates regulation in the fluid phase as well as on membranes by slowing down the amplification process by cleavage of C3-convertase and acting as a cofactor for *C*3*b*^*s*^ inactivation (27, 28). The ability of binding fluid phase Factor H and the resulting regulation of the complement cascade are the main reasons why host cells are usually not targeted by the complement system (26). As schematically shown in Figure 1A, our *DynaCoSys model* comprises all these features of *C*3^*f*^ and its derivatives: (i) activation, (ii) opsonization (iii) stabilization, (iv) amplification, and (v) regulation.

The molecule concentrations of the active *C*3*b*^*s*^ and inactive *iC*3*b*^*s*^ molecules at the cell surface are described by ODEs:

(1) $$\begin{array}{l} \frac{d}{dt}{C3b}^{s}=r_{ops}\cdot B_{C3b,free}\left({C3b}^{s},{iC3b}^{s}\right)\cdot {C3b}^{f}{\left(R\right)}^{*}\\ \;-\;r_{inhib}^{s}\left(fH^{s}\right)\cdot {C3b}^{s}, \end{array}$$

(2) $$\begin{array}{l} \frac{d}{dt}{iC3b}^{s}=r_{inhib}^{s}\left(fH^{s}\right)\cdot {C3b}^{s}-\;r_{dec}\cdot {iC3b}^{s} \end{array}$$

As can be seen in Equation (1), the dynamic increase of *C*3*b*^*s*^ molecules depends on the steady state concentration of soluble *C*3*b*^*f*^ (*R*)^*^ molecules at the cell surface, which are binding with rate *r*_*ops*_ to free binding sites at the cell surface that are present with concentration *B*_*C*3*b, free*_. *C*3*b*^*s*^ turns into its inactivated form *iC*3*b*^*s*^ with the rate $r_{inhib}^{s}$ – a process that is mediated by Factor H molecules at the cell surface that are present with concentration *fH*^*s*^. The decay of inactive *iC*3*b*^*s*^ molecules occurs spontaneously with rate *r*_*dec*_. All parameters used in this study are summarized in the Supplementary Material Section 2 and the analysis results of the steady state are given in the Supplementary Material Section 3. In particular, we show that tuning the *fH*^*s*^ concentration, the functions *C*3*b*^*s*^(*fH*^*s*^) and *iC*3*b*^*s*^(*fH*^*s*^) exhibit a removable singularity at Factor H concentration $fH_{th}^{s}=230\text{molecules}/\text{μ}m^{2}$, where the amplification process with rate *r*_*amp*_ and the inhibition process with rate $r_{inhib}^{s}$ of *C*3*b*^*s*^ balance out (*r*_*amp*_ = $r_{inhib}^{s}$; see Supplementary Figure S6). This threshold value coincides with the maximum change in all kinds of *C*3*b*^*s*^ molecule concentrations as a function of *fH*^*s*^ (see Supplementary Figure S7C) and corresponds to a relative usage of 0.41 % of all possible Factor H binding sites on the surface. Two regimes can be distinguished: for $fH^{s}<fH_{th}^{s}$ concentrations of *C*3*b*^*s*^ reach high values up to the complete coverage of the cell surface. We refer to this regime as the *non-self* regime; on the other hand, for $fH^{s}>fH_{th}^{s}$ only relatively low *C*3*b*^*s*^ concentrations are realized and we refer to this regime as *self* regime.

Modeling the interaction of cell surfaces with complement molecules in the fluid phase requires PDEs, because spatial concentration differences are expected in the surrounding volumes of activating surfaces. A simple decay model for *C*3*b*^*f*^ allows to estimate the interaction radius of a cell with these molecules in its environment (see Figure 1B). In this decay model, the active thioester bond *C*3*b*^*f*^ in aqueous solutions is assumed to be stabilized with a relatively short half-life time of 60 μ*s* by water molecules (29). This stabilization process renders molecules inactive for subsequent opsonization of cell surfaces. The volume in which activated molecules can diffuse before they are deactivated can be estimated by calculating the molecules' mean square displacement (see Supplementary Material Section 1). We find that the radius of a spherical volume around a cell where the concentration of *C*3*b*^*f*^ is still 1% of the typical value at the cell surface is not larger than 200 *nm* (see Figure 1B). In other words, *C*3*b*^*f*^ molecules that are able to reach a cell's surface are diffusing within a shell around the cell that is much smaller than the typical distance between cells in blood. For example, a concentration of 10^10^ *cells*/*l*, which is clearly above the cell count of typical infection scenarios in human blood, have a center-to-center distance of about 46 μ*m* (see Supplementary Material Section 1). It can be concluded that it is sufficient to consider a single cell in our hDE model. The reaction-diffusion equation for *C*3*b*^*f*^ is described by the PDE:

(3) $$\begin{array}{l} \frac{\partial {C3b}^{f}\left(r,t\right)}{\partial t}=D_{C3b}\cdot \Delta_{r}{C3b}^{f}\left(r,t\right)\\ \;-\left(r_{inhib}^{f}+\;r_{stab}\right)\cdot {C3b}^{f}{\left(r,t\right)}^{\;}+A \end{array}$$

with R ≤ r < ∞.

Here, *D*_*C*3*b*_ denotes the diffusion constant, *A* is the spontaneous *C*3*b*^*f*^ formation and the rates $r_{inhib}^{f}$ and *r*_*stab*_ refer to the inactivation of *C*3*b*^*f*^ (see Supplementary Material Section 1). The boundary condition at the cell surface (*r* = *R*) corresponds to Fick's first law, where the diffusion flux is calculated from the molecules that are formed at the surface (*r*_*amp*_) and bind (*r*_*ops*_) to the surface:

(4) $$\begin{array}{l} \frac{\partial {C3b}^{f}\left(r,t\right)}{\partial r}{|}_{r=R}\\ =\frac{r_{amp}\left(fH^{s}\right)\cdot {C3b}^{s}-\;r_{ops}\cdot B_{C3b,free}\left({C3b}^{s},{iC3b}^{s}\right)\cdot {C3b}^{f}\left(R\right)}{D_{C3b}} \end{array}$$

Far away from the cell surface (*r* → ∞) the boundary condition is given by:

(5) $$\begin{array}{l} {{C3b}^{f}\left(r\to \infty \right)}^{*}=\frac{A}{\;r_{inhib}^{f}+\;r_{stab}\;} \end{array}$$

As shown in detail in the Supplementary Material Section 1, it is appropriate to assume steady state conditions, i.e., solving Equation (3) for ∂*C*3*b*^*f*^ (*r, t*)/∂*t* = 0, because the reaction-diffusion dynamics equilibrates within milliseconds and by that much faster than the *C*3*b*^*s*^ dynamics on the cell surface.

**Figure 2:**
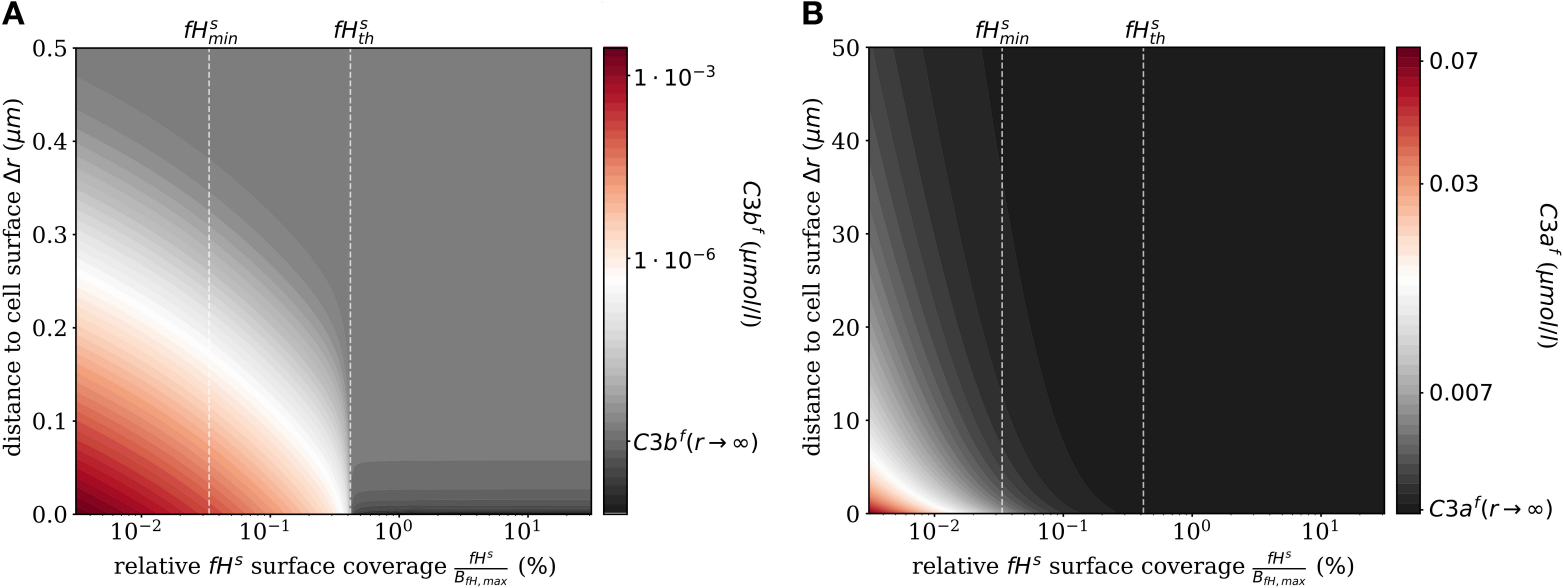
Steady states of the molecules *C3b*^*f*^ and *C3a*^*f*^ in the fluid phase for varying surface-bound Factor H concentrations. **(A)** Radial steady state concentration profile of the fluid-phase *C3b*^*f*^ concentration. **(B)** Radial steady state concentration profile of the fluid-phase *C3a*^*f*^ concentration. Mind the different scales in **(A,B)**.

The impact of surface-bound Factor H on the concentration of *C*3*b*^*f*^ in steady state is shown in Figure 2A as a function of the distance from the cell surface. As noted before, the threshold value $fH_{th}^{s}$ divides the cell surfaces into two regimes. For $fH^{s}<fH_{th}^{s}$, *C*3*b*^*f*^ molecules are formed to a larger extent than they are recruited and bound at the cell surface, and the opposite is true in the limit $fH^{s}>fH_{th}^{s}$. It follows that the net amount of diffused *C*3*b*^*f*^ increases with decreasing *fH*^*s*^ concentrations; for vanishingly small values of *fH*^*s*^, the concentration profile of *C*3*b*^*f*^ molecules decreases by almost six orders of magnitude. Regardless of the *fH*^*s*^ concentration, the *C*3*b*^*f*^ concentration profile reaches the equilibrium concentration at a distance from the cell surface of < 0.5 μ*m*. This distance is more than twice as large as estimated by our simple decay model (see Figure 1B), which yields a maximum distance of 0.2 μ*m*. However, this estimation did not include any cell surface, which restricts molecule diffusion to a half-space and by that effectively increases *C*3*b*^*f*^ concentration in the remaining volume. The obvious dependency of the spatial *C*3*b*^*f*^ concentration on *fH*^*s*^ confirms in retrospect that combining PDE and ODE in our hDE approach is required for a correct description of *C*3*b*^*f*^ concentrations on the cell surface as well as in the fluid phase.

Similarly, we compute the spatial distribution of *C*3*a*^*f*^ (see Figure 2B), which is affected by the amplification process but is not involved in cell surface opsonization. The steady state solution of the corresponding reaction-diffusion equation is given in Supplementary Material Section 1. The concentration of *C*3*a*^*f*^ molecules increases proportional to the concentration of C3-convertase molecules *C*3*b*^*s*^*Bb* and *C*3*b*^*s*^*BbP* with decreasing concentration of *fH*^*s*^. The relatively small *C*3*a*^*f*^ molecules diffuse up to 100 times further away from the cell surface than *C*3*b*^*f*^ molecules, due to a decay rate of *C*3*a*^*f*^molecules that is six orders of magnitude smaller than that of *C*3*b*^*f*^.

**Figure 3:**
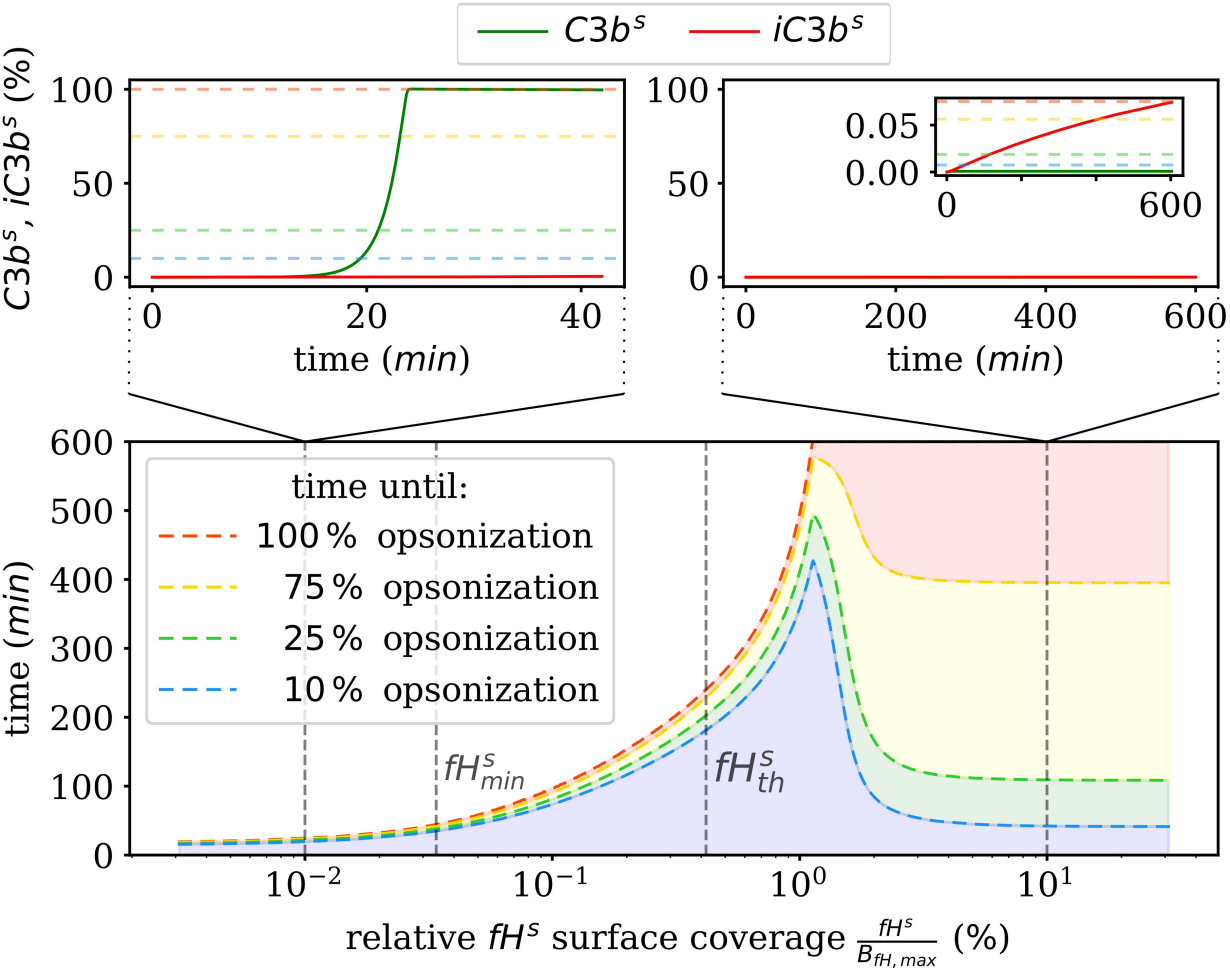
Summary of the numerical integration of 1,000 simulations for varied *fH*^*s*^ concentrations. The time intervals indicate how much time passes until a certain amount of *C3b*^*s*^ is attached to the cell surface. Two typical molecular concentration dynamics are shown for small *fH*^*s*^ concentrations (top left) and high *fH*^*s*^ concentrations (top right).

We also numerically integrate the dynamics of the hDE model for *C*3*b*^*s*^ and *iC*3*b*^*s*^ according to Equations (1, 2) as well as the dynamics of the amplification process on the surface as given in Supplementary Material Equations (S41–S44) of the Supplementary Material Section 1. This shows that the steady state is reached within times that are in agreement with the time scales of experimental data. The analysis of the time scales of all processes shows that, with the exception of the amplification process, they can be simplified within a quasi-steady state analysis (QSSA; see Supplementary Material Section 3). The resulting time scales are summarized in Figure 3, where it can be observed that increasing the *fH*^*s*^ concentration increases the time duration of opsonization. In the DynaCoSys model, cell surfaces with very low $fH^{s}<<\;fH_{th}^{s}$ concentrations exhibit the saturation of all binding sites by *C*3*b*^*s*^ molecules, i.e., about one million *C*3*b*^*s*^ molecules are bound at the cell surface within 5 min. Sheep erythrocytes, which activate the complement system because of a lack of factor H molecules (30, 31), get opsonized with more than one million *C*3*b*^*s*^ molecules within this time (29). Thus our model can reproduce the fast opsonization processes on complement activating surfaces. The corresponding opsonization dynamics follow a sigmoidal-shaped curve with a lag-phase of 15 min and a log phase of 5 min (see upper-left inset of Figure 3). Cell surfaces with $fH^{s}<fH_{th}^{s}$ concentrations reach the steady state in time durations comparable to a variety of pathogens, such as baker's yeast cells (~ 20 min) and *Escherichia coli* or *Staphylococcus aureus* (~120 min) (32). For *fH*^*s*^ concentrations close to $fH_{th}^{s}$ the complete opsonization process lasts about 200 min with a maintained ratio of lag to log phase. In the case $fH^{s}>fH_{th}^{s}$, the time duration of the opsonization process increases continuously and may be reached after only 6 h with vanishingly small *C*3*b*^*s*^ concentrations in steady state. Newly bound *C*3*b*^*s*^ molecules are immediately inactivated. Comparable dynamics are found for sheep erythrocytes (33), where the alternative pathway is not activated due to a high affinity to Factor H (30). Thus the time duration of our simulated data is in agreement with experimental observations. However, our simulated data for $fH^{s}<fH_{th}^{s}$ exhibits longer lag phases than experimental data, as for example in (32).

### Quantitative Prediction of Factor H Mediated Complement Evasion

**Figure 4:**
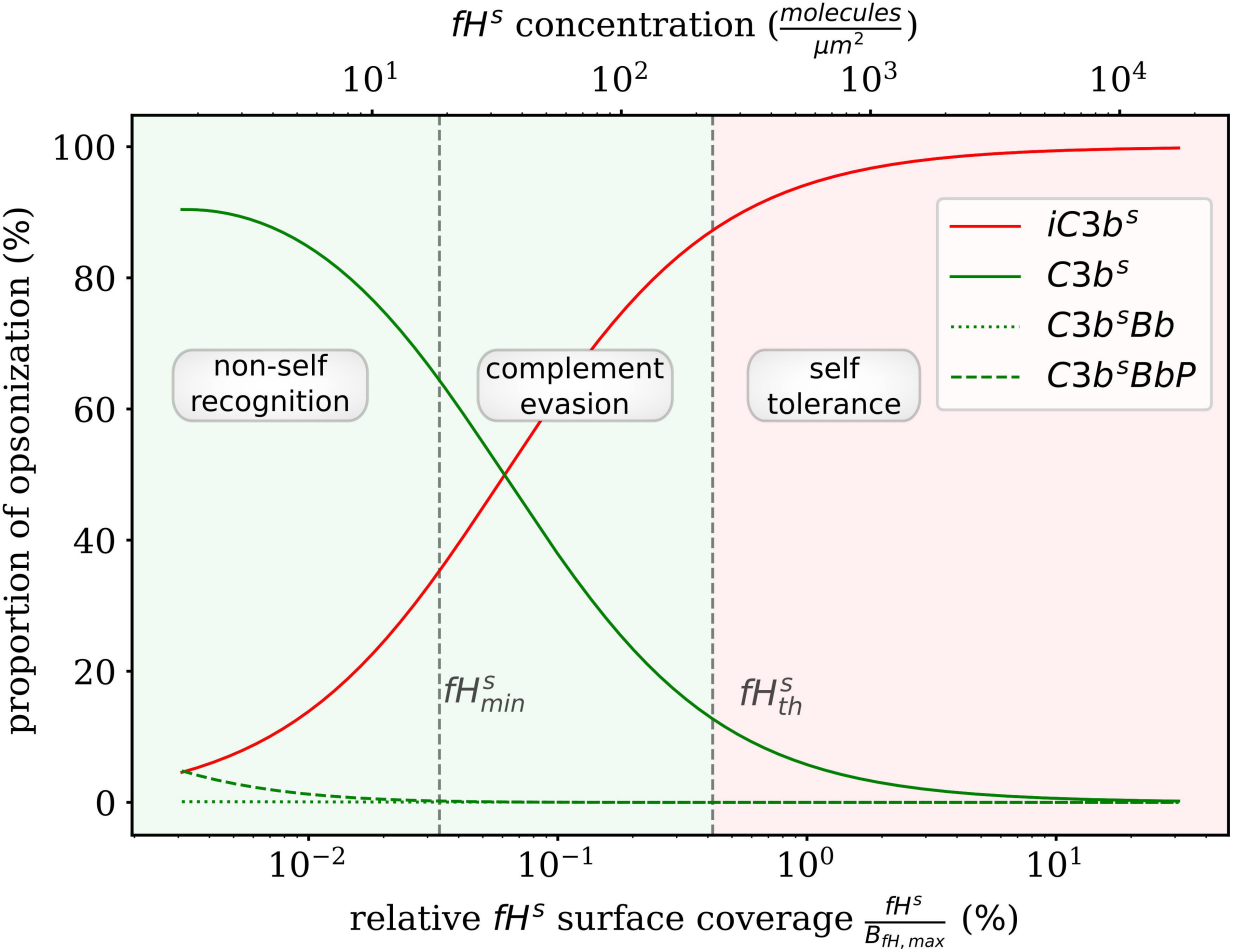
Proportion of *C*3*b* molecules at the cell surface. Based on the dominating types of *C*3*b* molecules, we distinguish the two extreme regimes for non-self recognition and self-tolerance that are separated by a transition regime where complement evasion takes place.

Complement evasion is mediated by the level of Factor H concentration at cell surfaces. The level of *fH*^*s*^ governs the opsonization of the cell surface by *C*3*b*^*s*^ and *iC*3*b*^*s*^ as well as the C3-convertase products *C*3*b*^*s*^*Bb* and *C*3*b*^*s*^*BbP*. Our mathematical hDE model enables us to quantify the existence of various regimes. In Figure 4, we plot the relative amount of these four molecule concentrations as a function of *fH*^*s*^ for the steady state solution of the *DynaCoSys model*. In the self-regime, the opsonization by *C*3*b*^*s*^ is strongly suppressed by Factor H yielding a high concentration of inactive derivative *iC*3*b*^*s*^. However, the non-self regime is subdivided into a regime where non-self recognition takes place and a regime where complement evasion is possible. The latter regime is an intermediate regime with $fH_{\min}^{s}\;<\;fH^{s}<fH_{th}^{s}$. The concentration $fH_{\min}^{s}$ is defined by the minimal number of Factor H molecules that is required that each point on the whole cell surface is within the radius of action of at least one Factor H molecule. This radius is defined by the length of the Factor H molecule chain of about 70 nm (3). Interestingly, our hDE model predicts that this Factor H concentration is associated with values of 70% > *C*3*b*^*s*^ > 10% as well as 30% < *iC*3*b*^*s*^ < 90%. We refer to this regime as “complement evasion” regime since comparable value ranges have been reported for immune evasive pathogens, such as *E. coli, S. aureus, Haemophilus influenzae, Streptococcus pneumoniae*, and *Streptococcus pyogenes* (32). Except for *S. aureus*, which provides its own membrane molecule taking over Factor H functionality (8, 14), all the other previously mentioned pathogens have been shown to evade complement via Factor H recruitment (16, 34–38). Furthermore, the non-self regime with low Factor H concentrations, $fH^{s}<fH_{\min}^{s}$, is associated with *C*3*b*^*s*^ concentration dominating *iC*3*b*^*s*^ concentration. In this limit, we also observe increased C3-convertase products *C*3*b*^*s*^*Bb* and *C*3*b*^*s*^*BbP* indicating the successful recognition of non-self. This increase in C3-convertase also induces a higher *C*3*b*^*f*^ (*R*) concentration at the cell surface (Figure 2A). This in turn reduces the duration of the opsonization process, which is up to one order of magnitude shorter compared to the regime of complement evasion (Figure 3). As can be seen in Figure 2B, in this regime the *C*3*a* concentration is increased accordingly by the higher C3-convertase.

**Figure 5:**
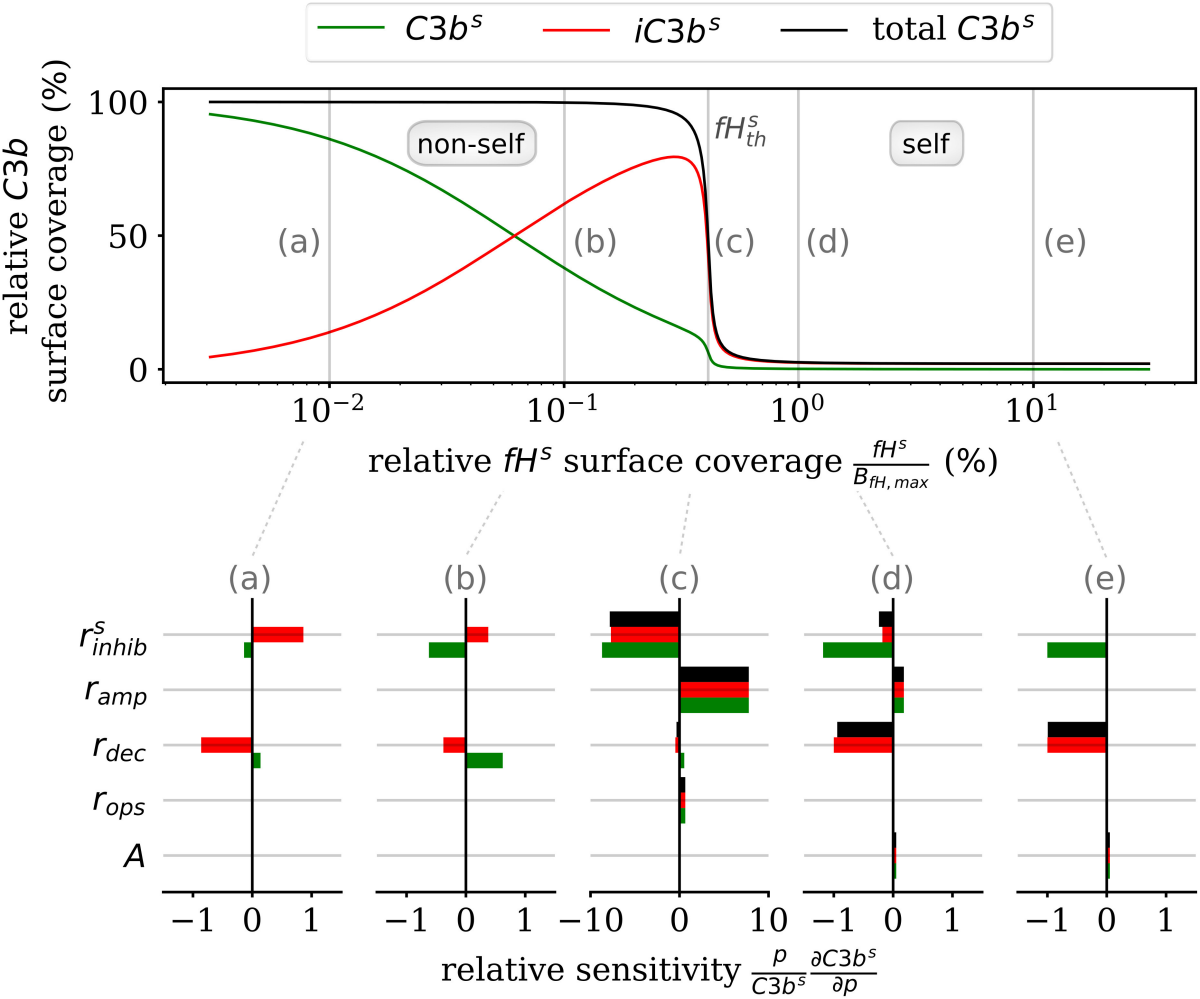
Sensitivity analysis of the model parameters for the steady state of surface-bound *C3b* molecules. The upper plot shows the relative concentration of complement molecules on the cell surface. The lower plots for selected Factor H concentrations give the relative local sensitivity of the complement molecules with respect to a parameter *p* that is individually varied. Positive sensitivities correlate with increasing molecule concentration. The non-self regime is sensitive to the rates *r*_*dec*_ and $r_{inhib}^{s}$ (see a,b). The transition region (c) is dominated by $r_{inhib}^{s}$ and *r*_*amp*_ and the self-regime is dominated by the parameters *A*, *r*_*dec*_, $r_{inhib}^{s}$, and *r*_*amp*_ (see d,e).

Furthermore, the *DynaCoSys model* enables us to dissect the individual impact of specific processes on the quantitative prediction of the opsonization level. In Figure 5, we plot the *C*3*b*^*s*^ surface coverage in steady state. Next, we performed a local sensitivity analysis by individually varying model parameters and monitoring their effects on the opsonization level as a function of *fH*^*s*^. The complete analysis is provided in the Supplementary Material Section 3, while Figure 5 summarizes the results for selected values of *fH*^*s*^ and for the following processes: (i) inhibition on surface with rate $r_{inhib}^{s}$ (see Supplementary Material Equation S32), (ii) amplification with rate *r*_*amp*_ (see Supplementary Material Equation S54), (iii) *iC*3*b*^*s*^ decay with rate *r*_*dec*_ (see Equation 2), (iv) opsonization with rate *r*_*ops*_ (see Supplementary Material Equation S13), and (v) spontaneous emergence of *C*3*b*^*f*^ with flux parameter *A* (see Supplementary Material Equation S11). The basic parameter values are given in the Supplementary Tables S10, S11 and in the Supplementary Figure S6.

In the non-self regime (see Figures 5a,b) the two rates $r_{inhib}^{s}$ and *r*_*dec*_ affect *C*3*b*^*s*^ (green) and *iC*3*b*^*s*^ (red) in an opposite fashion leaving the total opsonization level (black) unchanged. The total opsonization level in the non-self regime is generally quite constant, i.e., small changes in the model parameters do not have large effects on the close-to-maximal opsonization level. In contrast, the transition between the non-self and self regime (see Figure 5c) exhibits the highest relative sensitivities for all model parameters. The system is most sensitive to changes in the two model parameters $r_{inhib}^{s}$ and *r*_*amp*_, which underlines the importance of these two rates for the transition region. Both effective rates have approximately the same influence on the active as well as the inactive *C*3*b*^*s*^ molecules and this also affects the total opsonization level at $fH_{th}^{s}$. Interestingly, increasing *r*_*amp*_ will lead to a right-shift of the transition while increasing $r_{inhib}^{s}$ will lead to a left-shift of the transition region. The self-regime (see Figures 5d,e) is sensitive to the spontaneous activation *A*, the decay of the inactive *iC*3*b*^*s*^ molecules *r*_*dec*_ as well as $r_{inhib}^{s}$ and *r*_*amp*_. While the influence of amplification *r*_*amp*_ decreases with increasing *fH*^*s*^ concentration, the sensitivity to the spontaneous activation *A* is constant throughout the regime. In particular, for a relative Factor H surface coverage above *fH*^*s*^ = 1.72%, the spontaneous activation A is the only responsible factor for increasing *C*3*b*^*s*^ concentrations at the cell surface. In contrast, the non-self regime is not sensitive to the spontaneous activation A pointing toward opsonization that is induced by the amplification process at the cell surface. In general, while *C*3*b*^*s*^ and *iC*3*b*^*s*^ are similarly sensitive for *A* and *r*_*amp*_ and thus change the total opsonization level, the rates *r*_*dec*_ and $r_{inhib}^{s}$ change in addition the ratio between *C*3*b*^*s*^ and *iC*3*b*^*s*^. Finally, we find that the steady state of the cell surface concentrations does not depend on variations in the opsonization rate *r*_*ops*_. It can thus be concluded that this rate only affects the system dynamics.

### Complement Model Parameters Have Differential Impact on Critical Factor H Concentration

We investigate how the steady state of the DynaCoSys model behaves by screening various model parameters around their standard values given in Supplementary Tables S8–S11. Of particular interest are the molecule concentrations in the fluid phase, which can change depending on disease conditions as well as on medical treatments. The results are plotted for the concentration of all C3b molecules on the cell surface relative to the binding site concentration and as a function of the relative Factor H binding site concentration, i.e., the ratio of the concentration of binding sites for Factor H relative to its maximal concentration.

**Figure 6:**
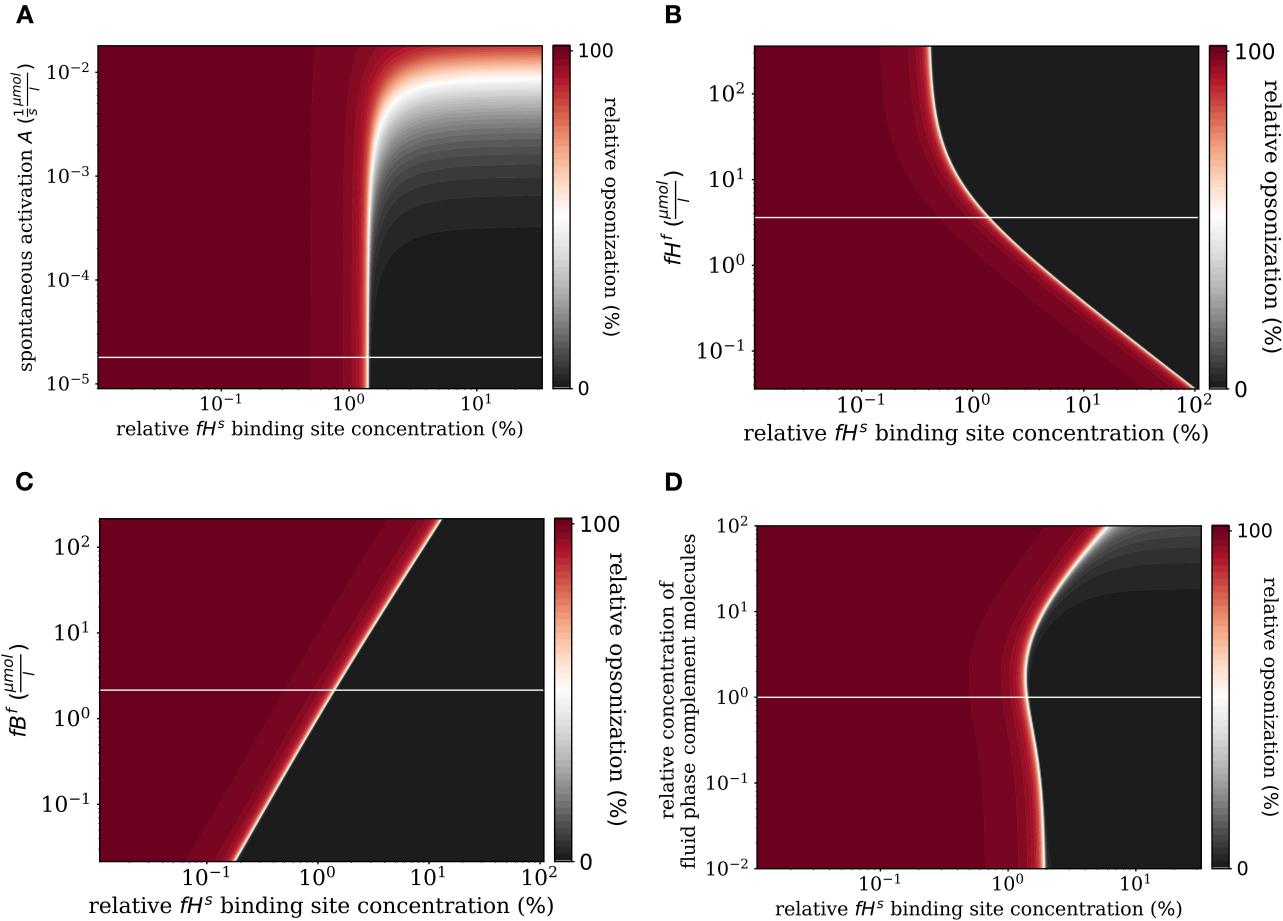
Relative opsonization in the steady state for varied system parameters as a function of the relative Factor H binding site concentration. The white line indicates the *DynaCoSys model* for standard parameter values. **(A)** Variation of the spontaneous *C3b*^*f*^ activation *A*, **(B)** variation of the *fH*^*f*^ concentration, **(C)** variation of the *fB*^*f*^ concentration, and **(D)** variation of the relative concentration of complement molecules in the fluid phase.

First, we varied the spontaneous *C*3*b*^*f*^ activation *A* (see Equation 3). Increasing this parameter affects only the self-regime by increasing the molecular concentration of all C3b molecules on the cell surface (Figure 6A). While the threshold value $fH_{th}^{s}$ remains the same, the self and non-self regime can no longer be distinguished for an increase of *A* by more than two orders of magnitude, since complete opsonization occurs in the self-regime. Furthermore, variation of *fH*^*f*^ affects the *fH*^*s*^ concentration on the cell surface (Figure 6B). With increasing *fH*^*f*^ concentration, more *fH*^*s*^ molecules bind, accelerating both the inhibition of *C*3*b*^*s*^, and the decay of the C3-convertase. For increased *fH*^*f*^ concentrations above one order of magnitude, *fH*^*s*^ becomes saturated. It follows that the threshold between the non-self and self regimes is shifted toward smaller relative Factor H binding sites concentrations, i.e., for higher *fH*^*f*^concentrations a cell is tolerated as self with less Factor H binding sites. In contrast, since the molecules *C*3^*f*^, Factor B (*fB*^*f*^), Factor D (*fD*^*f*^), and properdin (*fP*^*f*^) each enhance the amplification process positively (see Supplementary Material Sections 1, 3), increasing their concentrations is associated with shifts in $fH_{th}^{s}$ to higher relative Factor H binding sites concentrations. In particular, *fB*^*f*^ shows the largest effect of these molecules involved in the amplification process (see Figure 6C and Supplementary Material Section 3). Decreasing the fluid phase concentration, i.e., the concentration of each complement molecule, is often done in experiment to slow down the dynamics of the opsonization processes. In Figure 6D, we scan the behavior of the system for varied fluid phase concentrations. We find that an increased serum concentration leads to increased opsonization, which is characterized by a shift of $fH_{th}^{s}$ to higher binding site concentrations. The *DynaCoSys model* elucidates that, since above a certain *fH*^*f*^ concentration the Factor H binding sites on the cell surface are occupied, the *C*3*b*^*s*^ inactivation as well as cleavage of C3-convertase by Factor H are outcompeted by the amplification process. The decrease of the fluid phase concentration by up to two orders of magnitude exhibits a fairly constant threshold value $fH_{th}^{s}$.

**Figure 7:**
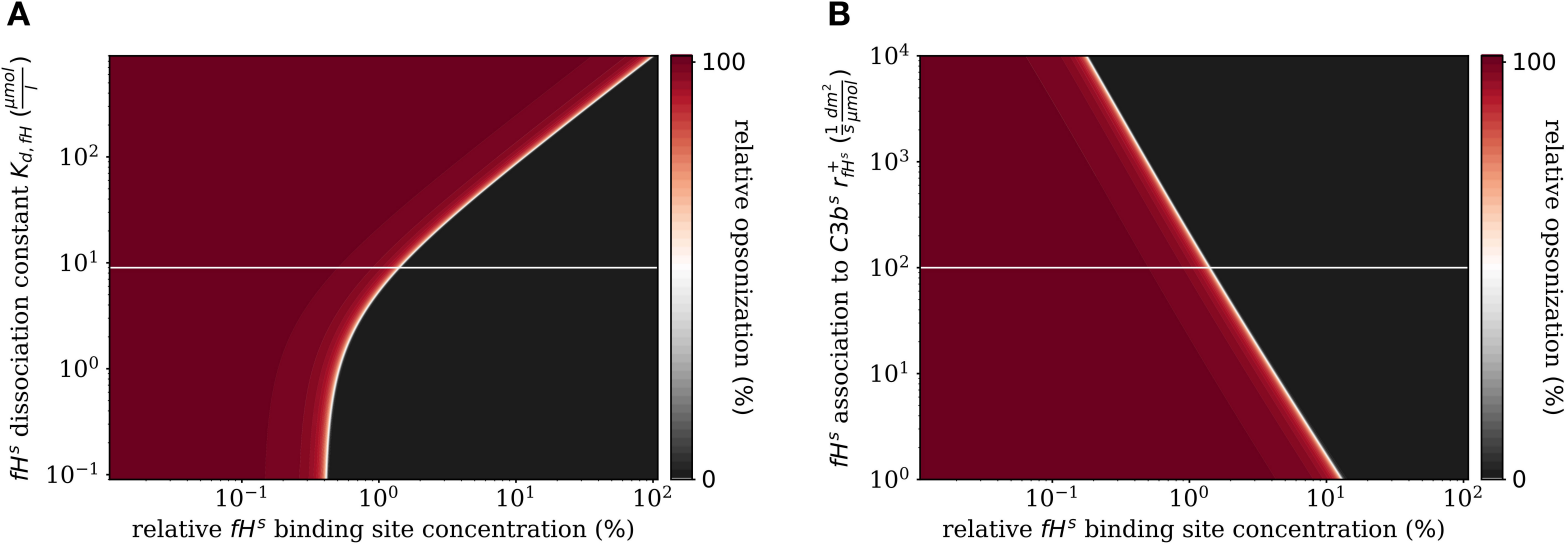
Relative opsonization in the steady state for varied system parameters as a function of the relative Factor H binding site concentration. The white line indicates the *DynaCoSys model* for standard parameter values. **(A)** Variation of the dissociation constant for Factor H and binding sites on the cell surface. **(B)** Variation of the reaction rate for *fH*^*s*^ binding to *C3b*^*s*^ molecules.

In addition to the variation of complement molecules, we here investigate the dissociation constant of Factor H and the cell surface (*K*_*d, fH*_, see Supplementary Material Section 1) and the reaction rate of *fH*^*s*^ binding to *C*3*b*^*s*^ ($r_{fH}^{+}$, see Supplementary Material Section 1). In the literature, both parameter values appear with large intervals of more than two orders of magnitude as well as quantitative variations depending on the type of cell surface (29). These also play a role in disease conditions caused by mutations that affect either cellular properties or the Factor H molecule binding to a cell surface (39, 40). For example, in patients with the atypic hemolytic uremic syndrome (aHUS) the dissociation constant *K*_*d,fH*_ and the binding rate $r_{fH}^{+}$ are, respectively, increased and decreased. The *DynaCoSys model* predicts that increasing the dissociation constant by two orders of magnitude leads to a complete opsonization of the cell surface regardless of the Factor H binding site concentration (Figure 7A). Thus, this implies that non-self and self cells alike will be attacked by the complement system establishing a permanent inflammatory milieu. Similarly, decreasing $r_{fH}^{+}$ by two orders of magnitude causes massive shift of $fH_{th}^{s}$ by a factor of ten to higher *fH*^*s*^ binding site concentrations (Figure 7B). We conclude that both the dissociation constant *K*_*d,fH*_ (Figure 7A) and the concentration of *fH*^*f*^ (Figure 6B) have a larger influence on the steady state than $r_{fH}^{+}$ if they are varied to the same extent.

## Discussion

In this study, we introduced the mathematical model *DynaCoSys* to simulate the dynamics of the complement system and to quantify the Factor H mediated self vs. non-self discrimination. The model focuses on the derivatives of the central complement molecule C3 and is based on a hybrid differential equation (hDE) approach, which combines ordinary differential equations (ODEs) and partial differential equations (PDEs) in one framework. To the best of our knowledge, *DynaCoSys* is the first model that models the dynamics on the cell surface using ODEs and using PDEs for the fluid phase that surrounds a cell. Previous models are typically based on ODEs only (19–21, 41–43) and do thus neglect the spatial distribution of *C*3*b* concentrations in the fluid phase. Compared to these models, which are trimmed for modeling details at the overwhelming costs of many parameters with large uncertainties in their unknown values (20, 21), the hDE approach of the *DynaCoSys model* allows reducing the number of model parameters by one order of magnitude. In particular, *DynaCoSys* involves only 35 model parameters of which the values for 34 parameters can be deduced from the literature (see Supplementary Tables S8–S11). The only remaining parameter that is unknown is the reaction rate of *fH*^*s*^ binding to *C*3*b*^*s*^ on the cell surface ($r_{fH}^{+})$, for which we applied a screening over 4 orders of magnitude (see Figure 7B). Furthermore, our model reveals that the concentration difference between the cell surface and the surrounding fluid phase covers six orders of magnitude confirming the importance of pursuing the hDE approach. Moreover, our analysis revealed for typical cell concentrations in blood, that these cells can be considered as independent with regard to the concentration *C*3*b*^*s*^ on cell surfaces and *C*3*b*^*f*^ in the fluid phase. In the future, *DynaCoSys* may be used as a starting point for modeling complement dynamics in aggregates of cells.

We applied a quasi-steady-state-approximation (QSSA) that enables reducing the full dynamics of the *DynaCoSys model* by five ODEs including twelve model parameters. These simplifications are made at the expense of some loss of accuracy in describing the complement dynamics. In the Supplementary Figure S9, we analyze the applicability of QSSAs and conclude that the QSSA for the dynamics of *C*3*b*^*f*^, the complement activation of the fluid phase as well as the regulation on the cell surface are well suited, because the time scales of these processes are at least one order of magnitude small than the time scale of the *C*3*b*^*s*^ dynamics. However, this argument does not hold for the amplification process, because its time scale is of the same order of magnitude compared to the *C*3*b*^*s*^ dynamics for $fH^{s}<fH_{th}^{s}$. In this case, the complete dynamics of the amplification process must be considered. By performing numerical simulations we showed that our model does provide quantitative results comparable to other models (20) and to experimental data. For example, complement activation in the non-self regime shows the typical sigmoidal shape with in the order of one million C3b molecules being bound within minutes as described by Pangburn et al. (29). In addition, the time durations for which the steady state is reached in the case of non-self cells are comparable to those measured for a variety of pathogens, such as *E. coli* or *S. aureus* (32). As expected, for the self-regime our model predicts significantly slower as well as lower opsonization due to the immediate deactivation of C3b on the cell surface by complement regulator Factor H.

Apart from these agreements between the mathematical model and experimental measurements, we also observe two differences. The first difference is with regard to the duration of the lag phase, which our simulations predict to be more extended than typically found in experiment. However, experiments also show that cells are very heterogenous regarding their C3b concentration during the lag phase (29). The lag phase is largely determined by the time it takes until the amplification loop on the cell surface has started (29). Spontaneously formed C3b molecules originating from the tick over process attach randomly on cell surfaces (5, 29), such that C3b amplification will start on some cells earlier than on others. This discrepancy can be explained by the fact that our hDE approach is based on differential equations and, thus, on the implicit assumption that molecules are present at high concentration levels. Similar deviations from experiment with regard to the lag phase are observed for the ODE-based model by Zewde et al. (20) supporting the interpretation that modeling the lag phase is inaccurate where only a few molecules exist on the cell surface. The limit of only a few molecules on the cell surfaces also affects the interaction of *C*3*b*^*s*^ and *fH*^*s*^, since the distribution of molecules are no longer representative for a homogeneous distribution on the cell surface. In order to model this limit correctly, a spatial agent-based approach may be used. However, an agent-based approach is associated with substantially higher computational load as well as additional parameters for the motion of each molecule. Another simplification of our model concerns the binding strength of Factor H, which for the sake of simplicity we set to a fixed value. Thus, even though the polymorphism of Factor H induces different binding strengths to the cell surface (27, 44), we considered the mean value of the associated distribution in terms of a constant dissociation constants (*K*_*d,fH*_). Taking a distribution for the binding strengths into account would broaden the relatively sharp transition between the self and non-self regime as a function of *fH*^*s*^ in the vicinity of $fH_{th}^{s}$ (see e.g., Figure 5, upper panel).

The second difference between the dynamics predicted by *DynaCoSys* and the dynamics in experiment is the abrupt saturation of the *C*3*b*^*s*^ concentration. On surfaces with high *C*3*b*^*s*^ concentrations, the C3-convertase molecule changes its affinity from a C3 substrate toward a C5 substrate (5, 45). Cleavage of C5 results into the molecules C5a and C5b. Here, the anaphylatoxin C5a is a proinflammatory protein that activates immune cells (5), while the fragment C5b initiates the terminal pathway of the complement system involving the formation of membrane attack complexes (MACs) on the cell surface (5). Associated with the C5 convertase, which is not part of the current version of *DynaCoSys*, less C3 molecules are cleaved and the *C*3*b*^*s*^ amplification loop slows down in experiment. In other words, our model overestimates the amplification of *C*3*b*^*s*^: while the flux of new *C*3*b*^*s*^ molecules is expected to decrease with increasing *C*3*b*^*s*^ concentration, the flux decreases abruptly when the surface is almost completely occupied. We decided against including the dynamics of the C5-convertase in the first version of the model, because—in contrast to the C3-convertase—no experimentally measured rates can be deduced from the literature. Related to this, opinions in the literature are divided about the affinity shifting of the convertase: in some studies it is concluded that an additional *C*3*b*^*s*^ molecule binds to the C3-convertase (46, 47), while other experiments seem to suggest that the increased concentration of *C*3*b*^*s*^ may cause a shift from the C3 substrate toward the C5 substrate (45). Since neither the reactions nor their rates can be unambiguously deduced from the literature, we decided against including the C5 cascade and the terminal pathway in a first version of the model.

In addition to C3b opsonization, complement modulates the immune response of the innate and adaptive immune system (3, 5). However, while the complement system responds to acute infections within a few minutes up to a maximum of a few hours, the innate and adaptive immune responses react on time scales of several hours and days to weeks, respectively. Therefore, the steady state of the complement system is of particular importance for the immune response on longer time scales. For this reason, models that study the interaction of immune cells with pathogens often consider the complement system to be in steady state (48, 49). Furthermore, the steady state offers the possibility to analyze predictions of the model analytically. While other models typically distinguish only between pathogens and host cells (20), the low computational effort of our analytical steady state solution allows for screening the whole space of unknown parameters, including also aspects like the binding site concentration of Factor H. The possibility to apply an in-depth screening enables identifying regimes by characteristic complement concentrations on the cell surface. Furthermore, we can observe transitions between such regimes and define thresholds derived from the analytical solution of our model in steady state. In this way, we identified the self-regime by nearly absent C3b molecules for high Factor H concentrations on the cell surface as well as the non-self regime, with an almost completely opsonized cells surface for relatively low Factor H concentrations. While the ratio of *iC*3*b*^*s*^ to *C*3*b*^*s*^ changes only slightly in the self-regime, a strong shift of this ratio is observed in the non-self regime. For pathogens this shift is of vital importance, even if the absolute number of complement molecules on the surface does not change. Non-active *iC*3*b*^*s*^ molecules imply a lower concentration of *C*3*a*^*f*^ and *C*3*b*^*f*^ molecules. As a consequence, the opsonization process is slower and the inflammatory milieu caused by *C*3*a*^*f*^ is much less pronounced, which increases the chance of the pathogens to survive the attack of the complement system. The transition between the regimes of non-self recognition and of self-tolerance is associated with the mathematically defined threshold $fH_{th}^{s}$, which marks the maximum change in the opsonization of the cell surface with respect to the Factor H concentration. Nevertheless, the transition between the non-self and self regimes is smooth and leaves room for an intermediate regime. In this regime, pathogens can evade the attack of the complement system by strongly lowering their opsonization level and by that protecting themselves from the immune response (50). On the other hand, self-cells can also fall below the threshold value $fH_{th}^{s}$ and become more recognizable for the complement system, e.g., dying cells undergoing apoptosis (5). Apoptosis is associated by the induction of specific intracellular pathways that also affect the cell surface (51) and is characterized by decreased Factor H concentrations (51) as well as a relatively large iC3b ratio (52), which renders apoptotic cells more recognizable by phagocytes for removal. Our sensitivity analysis revealed that the inhibition rate $r_{inhib}^{s}$, which is responsible for the inactivation of *C*3*b*^*s*^ to *iC*3*b*^*s*^, has a clear effect on surface molecule concentrations in both the non-self and self regime. This result supports the hypothesis that the recognition function of the complement system is in the interaction between surface-bound C3b and Factor H (28, 29, 50).

The *DynaCoSys model* of the complement system enables screening for the impact of various parameters and evaluating changes in the recognition of the cell surface in steady state. In particular, we can analyze pathological conditions that are directly associated with the complement system for three different reasons (24): (i) excessive complement activation exhausting the regular defense against complement damage, e.g., in the context of sepsis and immune complex disease (53, 54), (ii) lowered host defense against complement activation, e.g., syndromes like atypical hemolytic uremic syndrome (aHUS) that are caused by mutations (9), and (iii) altered host cells that activate the complement system, e.g., ischemia/reperfusion, burns, apoptotic/necrotic cells (55). The excessive complement activation can be associated with an increased *C*3*b*^*f*^ level. This can lead to an extreme increase in the opsonization level, even within the regime of self-tolerance. In particular, self-cells that are in close contact with complement-activating non-self cells during an immune response are exposed to this potential threat and may no longer be able to avoid their opsonization (56). This is another motivation to extend the *DynaCoSys model* to several interacting cells in contact, where also therapies that focus on the suppression of the activation of the alternative pathway may be simulated. Various approaches may be considered: (i) inhibition of C3 activation, (ii) inhibition of convertases, and (iii) inhibition of activating enzymes like Factor B or Factor D (12, 57). Our results show that the reduction of individual complement molecules, like Factor B, has a large impact on the opsonization of cell surfaces.

The treatment of diseases caused by immune evasive pathogenic microbes becomes more and more important due to increasing case numbers (58) and rising mortality (59). One of these pathogens with increasing case numbers is *Candida albicans*, which evades opsonization by the complement system through various mechanisms (60). Firstly, *C. albicans* can express several surface molecules with high affinity to Factor H (61). By including this effective increase of binding sites, the *DynaCoSys model* may be used to identify the most important binding sites. Secondly, *C. albicans* releases proteins that prevent the cleavage of C3 in the fluid phase and thus reduce C3b production in the vicinity of the cell surface (62). Our *DynaCoSys model* may be applied to monitor the dynamics in the fluid phase in order to elucidate this evasion mechanism. Taken together, the here established *DynaCoSys model* will be very well suited for detailed investigations of mechanisms of immune evasion in the future.

## Methods

The *DynaCoSys model* focuses on the three consecutive states of the central complement molecule *C*3*b*: the fluid phase molecule *C*3*b*^*f*^, the surface-bound molecule *C*3*b*^*s*^ and the inactivated surface-bound molecule *iC*3*b*^*s*^. The dynamics of other complement molecules are lumped into processes that are represented by effective rates under the following assumptions:

- Complement cofactor molecule concentrations can be considered as being constant in the fluid phase, because their consumption is negligibly small compared to their absolute concentrations. This reasonable assumption was also used in previous models of the complement system (20, 41, 42).
- The quasi steady state approximation can be applied to the dynamics of complement molecules with concentrations that equilibrate relatively fast compared to the main dynamics of the complement system (29, 63, 64).

In the next sections we summarize the main processes included in the *DynaCoSys model*. The model is described in full detail in the Supplementary Material Section 1. There we introduce the 37 reactions that are considered for the differential equations, we introduce the coupling between ODEs on the cell surface and PDEs in the fluid around the cell and we explain the simplifications that are done to obtain the Equations (1–5).

### Binding of Factor H to Cell Surface

The fluid-phase complement regulator Factor H is able to bind to binding sites at the cell surface (compare Figure 1A, orange box) (3, 9). Since the opsonization process of cell surfaces is characterized by a lag phase (33), it can be assumed that the Factor H concentration on the surface, *fH*^*s*^, is in a steady state. The concentration of surface-bound Factor H depends on the dissociation constant, *K*_*d,fH*_, the fluid phase concentration of Factor H, *fH*^*f*^, and the maximum concentration of binding sites, *B*_*fH*, max_, at the cell surface. The surface-bound Factor H concentration influences the Equations (1, 2, 4).

### Complement Activation in Fluid Phase

The activation of the alternative pathway takes place in the fluid phase and involves the spontaneous hydrolysis of *C*3^*f*^ (compare Figure 1A, blue box, activation). In the presence of the two co-Factor molecules Factor B (*fB*^*f*^) and Factor D (*fD*^*f*^) the initial C3-convertase *C*3(*H*2*O*)*Bb* is formed, which cleaves *C*3^*f*^ enzymatically into *C*3*b*^*f*^ and *C*3*a*^*f*^ (29, 65). The equilibrium concentration of the initial C3-convertase is proportional to the concentration of the precursor molecule *C*3^*f*^. Since *C*3^*f*^ is assumed to be constant in this model (see assumption i), there is a constant inflow of *C*3*b*^*f*^ molecules into the system given by the flux parameter *A*. The influx of *C*3*b*^*f*^ increases the *C*3*b*^*f*^ (*r*) concentration as described by Equations (3, 5).

### Binding of Fluid-Phase C3b to Cell Surface

Upon cleavage by C3-convertase, the *C*3*b*^*f*^ molecule exposes a highly reactive thioester bond (29, 66, 67). In aqueous solutions, this binding site has a very short half-life time of 60 μ*s* (29). The molecule can either bind to a cell surface with high affinity (24, 68) or it is inactivated, for example, by binding to a water molecule or by complement regulators (29). This opsonization process is modeled by the effective rate *r*_*ops*_ and is associated with the consumption of free binding sites *B*_*C*3*b,free*_ (Figure 1A, yellow box, opsonization). The opsonization process increases *C*3*b*^*s*^ (see Equation 1) and decreases the flux of *C*3*b*^*f*^ (see Equation 4). The concentration of free binding sites can be calculated using mass conservation considerations. Binding sites are occupied by active *C*3*b*, *C*3*b*^*s*^, intermediate products like C3-convertase molecules and inactive C3b: *iC*3*b*^*s*^. The inactivation process due to binding of a water molecule is modeled by an exponential decay with rate *r*_*stab*_ (Figure 1A, yellow box, stabilization) and appears in Equations (3, 5).

### Regulation of Active C3b Molecules

The regulation of the complement system takes place both in the fluid phase and on the cell surface. Factor H promotes the cleavage of *C*3*b*^*f*^ and *C*3*b*^*s*^ by Factor I into *C*3*b*^*f*^ and *C*3*b*^*s*^ via the same molecule cascade. The effective rates $r_{inhib}^{f}$ and $r_{inhib}^{s}$ summarize their dynamics, respectively, in the fluid and on the surface (Figure 1A, red box, regulation). This is accounted for by the surface dynamics in the Equations (1, 2) as well as by the fluid-phase dynamics in Equations (3, 5).

### Amplification of Surface-Bound C3b Molecules

The amplification mechanism includes the formation of C3-convertase based on surface-bound *C*3*b*^*s*^ molecules and the cleavage of *C*3^*f*^ molecules by the C3-convertase molecules resulting into the amplification of *C*3*b*^*f*^ concentration close to the cell surface. As in the fluid phase, *C*3*b*^*s*^ molecules react with Factor B and Factor D in order to form the C3-convertase molecule (29, 65). The formation of the C3-convertase is regulated by complement protein Factor H, which interferes sterically with C3-convertase complexes and accelerates the decay of these complexes (68, 69). The life time of a C3-convertase complex increases if properdin (*fP*^*f*^) binds to the complex (70, 71). It is also known to decelerate the C3-convertase decay, driven by Factor H (69, 71–73). Cleavage of the *C*3^*f*^ molecules to *C*3*b*^*f*^ and *C*3*a*^*f*^ is, as in the activation part, modeled by Michaelis-Menten kinetics. As the precursor molecules *C*3^*f*^ do not bind to the cell membrane, the nascent *C*3*b* molecule will belong to the fluid phase. The inflow of nascent *C*3*b*^*f*^ molecules at the cell surface is directly proportional to the *C*3*b*^*s*^ concentration with the effective rate *r*_*amp*_ (*fH*^*s*^), which enters (Equation 4) of the model.

## Data Availability Statement

The original contributions presented in the study are included in the article/Supplementary Material, further inquiries can be directed to the corresponding author/s.

## Author Contributions

AT and MTF conceived and designed this study, drafted the manuscript, revised it critically for important intellectual content, and final approval of the version to be published. MTF provided computational resources. Data processing, implementation and application of the computational algorithm were done by AT and TL. AT, TL, PZ, and MTF evaluated and analyzed the results of this study, and agree to be accountable for all aspects of the work in ensuring that questions related to the accuracy or integrity of any part of the work are appropriately investigated and resolved. All authors contributed to the article and approved the submitted version.

## Conflict of Interest

The authors declare that the research was conducted in the absence of any commercial or financial relationships that could be construed as a potential conflict of interest.
