# Supplementary material for: Quantification of Factor H Mediated Self vs. Non-self Discrimination by Mathematical Modeling
Source: Front Immunol. 2020 Sep 2;11:1911. doi: 10.3389/fimmu.2020.01911 (PMC7493836; doi:10.3389/fimmu.2020.01911)
Supplement: Supplementary file 1 [file Data_Sheet_1.pdf]

Supplementary Material

TABLE OF CONTENT

1 DynaCoSys model 2
1.1 Binding of Factor H to cell surface 2
1.2 Complement activation in fluid phase 4
1.3 Binding of fluid-phase $C3b$ to cell surface 6
1.4 Regulation of active $C3b$ molecules 7
1.5 Amplification of surface-bound $C3b$ molecules 11
1.6 Spatial distribution of fluid phase $C3b$ molecules 15
2 Parameters of the DynaCoSys model 20
2.1 Model parameters 20
3 Analysis of the DynaCoSys model 24
3.1 Complement dynamics of C3b-opsonization requires hybrid differential equation approach 24
3.2 Quantitative prediction of Factor H mediated complement evasion 28
3.3 Complement model parameters have differential impact on critical Factor-H concentration 30

1 DYNACOSYS MODEL

In the following section, the DynaCoSys model will be discussed in detail. Each feature of the model is shown in **Supplementary Figure S1** in a separate colored box and will be discussed in a separate subsection.

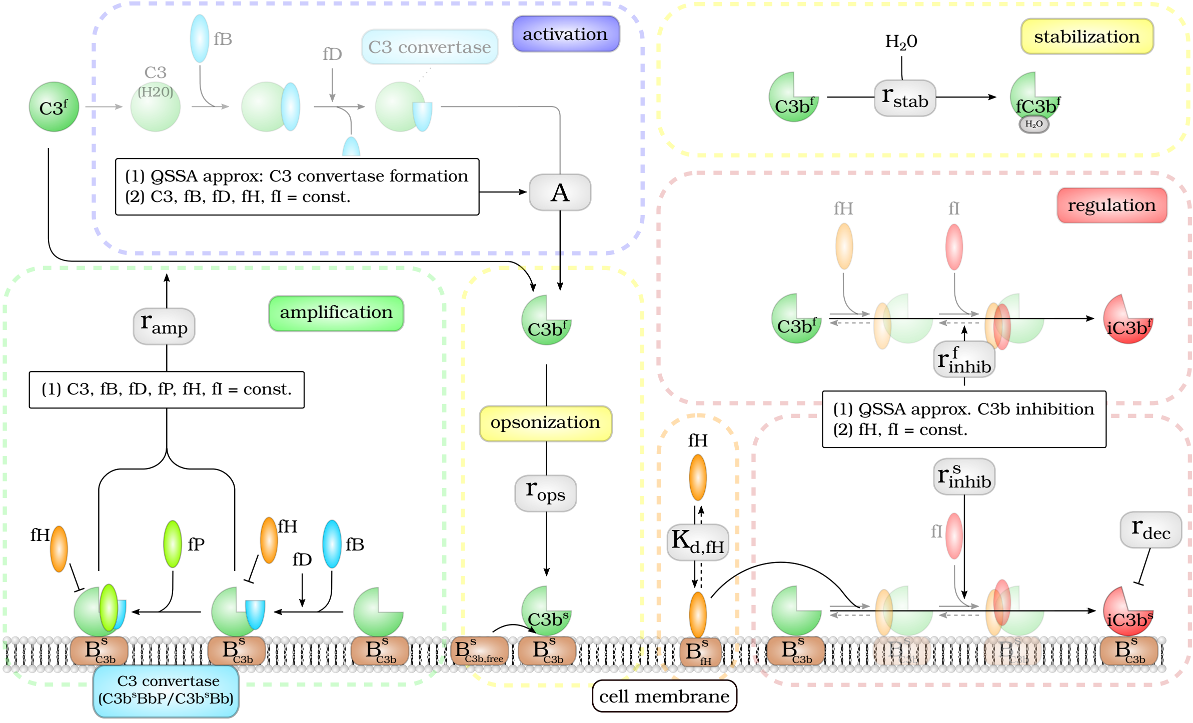

Figure S1. Model of the complement system. Complement activation can be divided into four parts: activation, opsonization and stabilization, as well as regulation and amplification. The model focuses on the dynamics of the central component $C3b$: Active $C3b$ in the fluid phase, $C3b^f$, results from cleavage of precursor molecule $C3^f$. The interaction of the fluid phase molecule $C3b^f$ with the cell surface is modeled by the interaction with free surface binding sites B^* and binding sites B that are occupied with molecules $C3b^s$ on the surface. Active $C3b^s$ can form a cell-bound C3-convertase molecule that cleaves further $C3^f$ molecules to $C3b^f$ molecules in the vicinity of the cell surface. Active $C3b^s$ can be inactivated via a Factor H mediated inhibition process, while active $C3b^f$ that does not bind to the cell surface gets inactivated via a Factor H mediated inhibition process and is no longer able to bind to the cell surface.

1.1 Binding of Factor H to cell surface

In the presence of suitable binding sites, the fluid phase complement regulator Factor H is able to bind to surfaces [1, 2]. We model a reversible binding process as shown in **Supplementary Table S1**. The orange box of **Supplementary Figure S1** illustrates this process. Using the law of mass action, the reactions are translated into a system of ordinary differential equations, with $B^s_{fH,free}$ giving the concentration of free Factor H binding sites, and fH^f and fH^s denoting the concentration of Factor H molecules in the fluid phase and on the surface, respectively:

$$\frac{d}{dt} fH^f = -r_{fH^f \to fH^s} \cdot fH^f \cdot B^s_{fH,free} + r_{fH^s \to fH^f} \cdot fH^s, \tag{S1}$$

Table S1. Biochemical reactions of the attachment process of Factor H to surfaces

(1)	$fH^f + B^s_{fH,free} \underset{r_{fH^s \to fH^f}}{\overset{r_{fH^f \to fH^s}}{\rightleftarrows}} fH^s$	Factor H attaching/detaching to surface

$$\frac{d}{dt} fH^s = r_{fH^f \to fH^s} \cdot fH^f \cdot B^s_{fH,free} - r_{fH^s \to fH^f} \cdot fH^s. \tag{S2}$$

The concentration of free binding sites is limited by the maximum concentration of binding sites, $B_{tot,fH}$, which find space on the cell surface. The upper limit of binding sites is estimated by assuming a monolayer of Factor H molecules binding with SCR-19/20 [3] on the cell surface. The area covered by the SCR-19/20 domain is approximately $20\,nm^2$ [4] yielding a maximum concentration of fH binding sites to be $B_{fH,max} = 5.56 \cdot 10^4 \frac{molecules}{\mu m^2}$. This maximum concentration is purely theoretically motivated and is used in the following to normalize the actual fH^s concentration. Using molecule conservation considerations we can calculate the concentration of free binding sites by

$$B^s_{fH,free} = B^s_{fH,max-cell} - fH^s. \tag{S3}$$

The concentration $B^s_{fH,max-cell}$ characterizes the concentration of Factor H binding sites on a cell and is always smaller than the theoretical maximum concentration $B_{fH,max}$.

Since the opsonization process of cell surfaces is characterized by a lag phase [5], it can be assumed that the Factor H concentration on the surface, fH^s, is in a steady state before relevant amounts of C3b molecules bind on the cell surface. The steady state concentration of the surface bound Factor H molecules is given by

$$fH^s = B^s_{fH,max-cell} \cdot \frac{fH^f}{K_{d,fH} + fH^f}. \tag{S4}$$

The concentration of surface-bound Factor H depends on the serum parameter fH^f and the two cell surface parameters: (i) the dissociation constant,

$$K_{d,fH} = \frac{r_{fH^s \to fH^f}}{r_{fH^f \to fH^s}}, \tag{S5}$$

and (ii) the concentration of binding sites $B^s_{fH,max-cell}$ on the cell surface. Another important parameter is the concentration of Factor H molecules fH^s_{min} that is at least required to completely cover the entire cell surface. It is assumed that Factor H can control a hemisphere of $70\,nm$ [1]. Thus, the concentration of $19\frac{molecules}{\mu m^2}$ Factor H molecules is at least necessary for a complete control of the surface, which corresponds to approximately $0.03\,\%$ of the maximum possible Factor H binding sites.

This study investigates how the fH^s concentration affects the opsonization of the cell. Therefore, it is necessary to vary the Factor H concentration. This requires that the three parameters fH^f, $K_{d,fH}$ and $B_{fH,max-cell}$ are varied in order to manipulate the fH^s concentration. In this study we will tune the concentration of binding sites, since there is a linear relationship to the fH^s concentration.

1.2 Complement activation in fluid phase

The activation of the complement system's alternative pathway takes place in the fluid and includes the spontaneous hydrolysis of $C3^f$. The resulting $C3(H2O)$ is structurally different from $C3b$ [6], but has the same properties [7]. It offers a binding site for the complement Factor B (fB^f) which is subsequently cleaved by Factor D (fD^f). The resulting complex is the initial C3-convertase $C3(H2O)Bb$, which cleaves $C3^f$ enzymatically into $C3b^f$ and $C3a^f$. The initial convertase has a half-life time of $77\,s^{-1}$ [8], which is further reduced by the presence of Factor H [9, 10]. Factor H also offers a binding site for Factor I [11], which cleaves $C3b$ and $C3(H2O)$ molecules, and therefore an inactivation process is added. The described biochemical reactions are summarized in **Supplementary Table S2** and illustrated in **Supplementary Figure S1** in the blue box.

Table S2. Biochemical reactions involved in the formation of inital C3 convertase.

	Hydrolysis	
(1)	$C3^f \xrightarrow{r^+_{C3^f(H_2O)}} C3^f(H_2O)$	Hydrolysis of $C3^f$ [7, 12]
	Formation of initial concertase	
(2)	$C3^f(H_2O) + fB^f \xrightarrow{r^+_{C3^f(H_2O)B}} C3^f(H_2O)B$	Attachament of Factor B [7, 12]
(3)	$C3^f(H_2O)B \xrightarrow{r_{C3^f(H_2O)B,cleave},\, fD^f} C3^f(H_2O)Bb + Ba^f$	Enzymatically cleavage of Factor B [7, 12]
(4)	$C3^f(H_2O)B \xrightarrow{r^-_{C3^f(H_2O)B}} C3^f(H_2O) + fB^f$	Spontaneous decay of $C3^f(H_2O)B$ [7, 12]
(5)	$C3^f(H_2O)Bb \xrightarrow{r^-_{C3^f(H_2O)Bb}} C3^f(H_2O) + Bb^f$	Spontaneous decay of $C3^f(H_2O)Bb$ [7, 12]
(6)	$C3^f(H_2O)Bb + fH^f \xrightarrow{r^-_{C3^f(H_2O)Bb,fH}} C3^f(H_2O) + fH^f + Bb^f$	Factor H assisted decay of $C3^f(H_2O)Bb$ [7, 12]
	Inactivation	
(7)	$C3^f(H_2O) + fH^f \xrightarrow{r^+_{C3^f(H_2O),fH}} C3^f(H_2O)H$	Factor H binding to $C3^f(H2O)$ [7, 13]
(8)	$C3^f(H_2O)H \xrightarrow{r^-_{C3^f(H_2O),fH}} C3^f(H_2O) + fH^f$	release of Factor H from $C3^f(H2O)H$ [7, 13]
(9)	$C3^f(H_2O)H \xrightarrow{r_{C3^f(H_2O),inhib},\, fI^f} iC3^f(H_2O) + fH^f$	Enzymatically cleavage of f $C3^f(H2O)$ [7, 13]

The reactions are transferred to the following set of ordinary differential equations using the law of mass action. Additionally, the concentrations of the fluid phase complement proteins $C3^f$, fB^f, fD^f, fP^f,

fH^f and fI^f are assumed to be constant, since the molecules are available in excess [14, 15, 16]:

$$\begin{aligned}\frac{d}{dt}C3^f(H_2O) = &-r^+_{C3^f(H_2O)B} \cdot fB^f \cdot C3^f(H_2O) - r^+_{C3^f(H_2O),fH} \cdot fH^f \cdot C3^f(H_2O) \\ &+ r^+_{C3^f(H_2O)} \cdot C3^l + r^-_{C3^f(H_2O)B} \cdot C3^f(H_2O)B + r^-_{C3^f(H_2O)Bb} \cdot C3^f(H_2O)Bb \\ &+ r^-_{C3^f(H_2O)Bb,fH} \cdot fH^f \cdot C3^f(H_2O)Bb + r^-_{C3^f(H_2O),fH} \cdot C3^f(H_2O)H,\end{aligned} \tag{S6}$$

$$\begin{aligned}\frac{d}{dt}C3^f(H_2O)B = &\, r^+_{C3^f(H_2O)B} \cdot fB^f \cdot C3^f(H_2O) \\ &- r_{C3^f(H_2O)B,cleave}\frac{fD^f \cdot C3^f(H_2O)B}{K_d + fD^f} - r^-_{C3^f(H_2O)B} \cdot C3^f(H_2O)B,\end{aligned} \tag{S7}$$

$$\begin{aligned}\frac{d}{dt}C3^f(H_2O)Bb = &\, r_{C3^f(H_2O)B,cleave}\frac{fD^f \cdot C3^f(H_2O)B}{K_d + fD^f} \\ &- r^-_{C3^f(H_2O)Bb} \cdot C3^f(H_2O)Bb - r^-_{C3^f(H_2O)Bb,fH} \cdot fH^f \cdot C3^f(H_2O)Bb,\end{aligned} \tag{S8}$$

$$\begin{aligned}\frac{d}{dt}C3^f(H_2O)H = &-r^-_{C3^f(H_2O),fH} \cdot C3^f(H_2O)H - r_{C3^f(H_2O),inhib}\frac{fI^f \cdot C3^f(H_2O)H}{K_d + fI^f} \\ &+ r^+_{C3^f(H_2O),fH} \cdot fH^f \cdot C3^f(H_2O).\end{aligned} \tag{S9}$$

The steady state of the initial C3 convertase, the equilibrium concentration of the inital C3-convertase, is proportional to the concentration of the precursor molecule $C3^f$:

$$\begin{aligned}C3^f(H_2O)Bb^* = &\frac{r_{C3^f(H_2O)B,cleave}\frac{fD^f}{K_d+fD^f}}{r^-_{C3^f(H_2O)Bb} + r^-_{C3^f(H_2O)Bb,fH} \cdot fH^f} \\ &\cdot \frac{r^+_{C3^f(H_2O)B} \cdot fB^f}{r^-_{C3^f(H_2O)B} + r_{C3^f(H_2O)B,cleave}\frac{fD^f}{K_d+fD^f}} \\ &\cdot \frac{r^+_{C3^f(H_2O)}\left(r^-_{C3^f(H_2O),fH} + r_{C3^f(H_2O),inhib}\frac{fI^f}{K_d+fI^f}\right)}{r_{C3^f(H_2O),inhib}\frac{fI^f}{K_d+fI^f}} \cdot C3^f.\end{aligned} \tag{S10}$$

We assume that the blood plasma is already in equilibrium before the experiment begins. Thus we will use the steady state solution of the inital C3-convertase to calculate the inflow of $C3b^f$ molecules, that arise as a result of spontaneous activation.

The cleavage reaction of $C3^f$ is an enzymatic reaction in which the C3 convertase $C3^f(H2O)Bb$ cleaves $C3^f$ into $C3a^f$ and $C3b^f$. This enzymatic reaction is modeled with Michaelis-Menten kinetics using the

Briggs-Haldane approxiamtion. The ODE based on the Michaelis-Menten kinetics is given by:

$$\frac{d}{dt}C3b^f_{activation} = r_{init\_cleav.} \cdot C3^f(H_2O)Bb^* \frac{C3^f}{K_{m,act} + C3^f} = A = const., \tag{S11}$$

$$K_{m,act} = \frac{r^-_{conv.} + r_{init\_cleav.}}{r^+_{conv.}}. \tag{S12}$$

The influx resulting from spontaneous activation is constant, since $C3^f$ as well as the steady state of the inital C3-convertase are constant, so that there is a constant flow of $C3b^f$ molecules in the system with the flow rate A.

Many parameters are involved in describing the steady state concentration of the initial C3-convertase $C3^f(H_2O)Bb^*$. Most of the parameters can be take from the literature for the homologeous molecule C3b. However, since there is a certain inaccuracy in the transfer of the values, the constant inflow of C3b molecules is determined using experimental data. Based on experiments by Pangburn *et al.* C3 is consumed by $1\,\%/h$ under physiological conditions [17]. From these experiments a constant inflow of $A = 1.8 \cdot 10^{-5}\,\frac{1}{s}\frac{\mu mol}{l}$, the spontaneous activation, is calculated [17]. With the help of the **Supplementary Equations S11** and **S10** we estimate the constant inflow for non physiological conditions using a linear relationship between $C3^f$ and $C3^f(H_2O)Bb^*$.

1.3 Binding of fluid-phase $C3b$ to cell surface

Upon cleavage by C3-convertase, $C3b^f$ is able to bind to the cell surface. The cleavage process leads to the exposition of a highly reactive thioester bond [6, 12]. In aqueous solutions, this binding site has a very short half-life time of $60\,\mu s$ [7]. The molecule can either bind to a cellular surface with high affinity [18, 19] or it is inactivated, for example, by binding to a water molecule or by complement regulators. This opsonization process is modeled by the rate r_{ops} and is associated with the consumption of free binding sites $B^s_{C3b,free}$. The reaction is given in **Supplementary Table S3** and visualized in the yellow box (opsonization) in **Supplementary Figure S1**.

Table S3. Biochemical reactions of the opsonization process of C3b to surfaces

(1)	$C3b^f(R) + B^s_{C3b,free} \xrightarrow{r_{ops}} C3b^s$	C3b attaching to surface

The resulting ODE is given by

$$\frac{d}{dt}C3b^s_{opsonization} = r_{ops} \cdot B^s_{C3b,free} \cdot C3b^f(R). \tag{S13}$$

Since a covalent bond to the surface is formed [18], we do not model an off-reaction. The concentration of free binding sites $B^s_{C3b,free}$ can be calculated using mass conservation considerations. Binding sites are occupied by active $C3b$, $C3b^s$, intermediate products like C3-convertase molecules and inactive $C3b$: $iC3b^s$. We calculate the maximum concentration of C3b binding sites $B^s_{C3b,tot}$ under the assumption of a

closed monolayer of $C3b^s$ molecules. The C3b molecule was oriented on the surface so that the thioester bond is in contact with the surface. According to the protein data base, the resulting contact area between molecule and cell surface is $8.25 \cdot 10^{-5}\,\mu m$ [5, 20, 21]. The resulting maximum concentration of C3b binding sites is given by $B^s_{C3b,tot} = 1.21 \cdot 10^4\,\mu m^{-2}$.

The inactivation process due to binding of a water molecule is modeled by an exponential decay with rate r_{stab}. The reaction is given in **Supplementary Table S4**, visualized in the yellow box (stabilization) in **Supplementary Figure S1**.

Table S4. Biochemical reactions of the stabilization process of $C3b^f$ in the fluid

(1)	$C3b^f \xrightarrow{r_{stab}} C3b^f_{stab}$	$C3b^f$ stabilization

The resulting ODE is given by

$$\frac{d}{dt}C3b^f_{stabilization} = -r_{stab} \cdot C3b^f. \quad \text{(S14)}$$

The stabilized $C3b^f$ molecule is no longer able to bind to surfaces and will not be part of our further analysis.

1.4 Regulation of active $C3b$ molecules

The inhibition process, the cleavage of $C3b$ by Factor I to $iC3b$, has the same mechanisms in the fluid and on the cell surface [22]. Factor H binds reversibly to $C3b$ and forms a $C3bH$ complex. Factor I binds reversible to the $C3bH$ complex forming a $C3bH, I$ complex in which $C3b$ is cleaved enzymatically. The reactions that take place in that process are given in **Supplementary Table S5** and are visualized in the red box of **Supplementary Figure S1**.

Table S5. Biochemical reactions of the regulation of C3b in the fluid and on the surface

(1)	$C3b^{f/s} + fH^{f/s} \underset{r^{-,f/s}_{fH}}{\overset{r^{+,f/s}_{fH}}{\rightleftharpoons}} C3b^{f/s}H$	Factor H attaching/detaching to $C3b$ [22]
(2)	$C3b^{f/s}H + fI^f \underset{r^{-,f/s}_{fI}}{\overset{r^{+,f/s}_{fI}}{\rightleftharpoons}} C3b^{f/s}H, I$	Factor I attaching/detaching to $C3bH$ [22]
(3)	$C3b^{f/s}H, I \xrightarrow{r^{f/s}_{cleave}} iC3b^{f/s} + fH^{f/s} + fI^f$	cleavage of the $C3b$ molecule [22]

These reactions can be translated by the law of mass action to the following set of differential equations:

$$\frac{d}{dt}C3b^{f/s} = -r^{+,f/s}_{fH} \cdot fH^{f/s}_{free} \cdot C3b^{f/s} + r^{-,f/s}_{fH} \cdot C3b^{f/s}H, \quad \text{(S15)}$$

$$\frac{d}{dt}C3b^{f/s}H = r_{fH}^{+,f/s} \cdot fH_{free}^{f/s} \cdot C3b^{f/s} - r_{fH}^{-,f/s} \cdot C3b^{f/s}H \\ - r_{fI}^{+,f/s} \cdot fI^{f} \cdot C3b^{f/s}H + r_{fI}^{-,f/s} \cdot C3b^{f/s}H, I, \tag{S16}$$

$$\frac{d}{dt}C3b^{f/s}H, I = r_{fI}^{+,f/s} \cdot fI^{f} \cdot C3b^{f/s}H - r_{fI}^{-,f/s} \cdot C3b^{f/s}H, I - r_{cleave}^{f/s} \cdot C3b^{f/s}H, I, \tag{S17}$$

$$\frac{d}{dt}iC3b^{f/s} = r_{cleave}^{f/s} \cdot C3b^{f/s}H, I. \tag{S18}$$

The fluid phase molecules fI^{f} is present in large concentrations. It is assumed that its concentration is not significantly reduced by the formation of the complexes $C3b^{f}H, I$. Thus, the concentration of unbound molecules fI_{free}^{f} is approximately the same as the maximum number of molecules in the fluid:

$$fI^{f} = fI_{free}^{f} + C3b^{f/s}H, I \approx fI_{free}^{f}. \tag{S19}$$

The steady state of the $C3bH, I$ concentration, $C3b^{f/s}H, I^{*}$, can be calculated from **Supplementary Equation S17** and is given by

$$C3b^{f/s}H, I^{*} = \frac{fI^{f/s}}{K_{m,I}^{f/s}} \cdot C3b^{f/s}H^{*}, \tag{S20}$$

$$K_{m,I}^{f/s} = \frac{r_{fI}^{-,f/s} + r_{cleave}^{f/s}}{r_{fI}^{+,f/s}}. \tag{S21}$$

In order to calculate the steady state of the $C3bH$ concentration, $C3b^{f/s}H^{*}$, we have to analyze the concentration of free Factor H binding sites $fH_{free}^{f/s}$. In the fluid phase, we add an analogous approximation of the free Factor H molecules fH_{free}^{f} as we already did for the Factor I molecules in **Supplementary Equation S19**:

$$fH^{f} = fH_{free}^{f} + C3b^{f}H + C3b^{f}H, I \approx fH_{free}^{f}. \tag{S22}$$

The $C3b^{f}H^{*}$ steady state concentration follows from the **Supplementary Equations S16** and **S20** and is given by

$$C3b^{f}H^{*} = \frac{r_{fH}^{+,f} \cdot \frac{K_{m,I}^{f}}{r_{cleave}^{f}} \cdot fH^{f} \cdot C3b^{f^{*}}}{K_{inhib}^{f} + fI^{f}}, \tag{S23}$$

$$K^f_{inhib} = \frac{K^f_{m,I} \cdot r^{-,f}_{fH}}{r^f_{cleave}}. \tag{S24}$$

On cell surfaces, the concentration of Factor H molecules is not inexhaustible. Thus, the number of surface bound molecules, which are able to bind to $C3b$ molecules, might be smaller than the maximum number of surface bound Factor H molecules. The concentration fH^s_{free} describes the concentration of surface bound Factor H that forms no complexes on the surface and is given by

$$fH^s_{free} = fH^s - C3b^sH - C3b^sH, I \tag{S25}$$

using the conservation law. The steady state solution of $C3b^sH^*$ can be calculated from the **Supplementary Equations S16** and **S20** and is given by

$$C3b^sH^* = \frac{r^{+,s}_{fH} \cdot \frac{K^s_{m,I}}{r^s_{cleave}} \cdot fH^s \cdot C3b^{s*}}{r^{+,s}_{fH} \cdot C3b^{s*} \left(\frac{K^s_{m,I} + fI^f}{r^s_{cleave}}\right) + K^s_{inhib} + fI^f}, \tag{S26}$$

$$K^s_{inhib} = \frac{K^s_{m,I} \cdot r^{-,s}_{fH}}{r^s_{cleave}}. \tag{S27}$$

The concentration of free surface bound Factor H molecules fH^s_{free} can be calculated from the conservation law in **Supplementary Equation S25** and the steady states in **Supplementary Equation S20** and **S26**:

$$fH^s_{free} = fH^s \frac{K^s_{inhib} + fI^f}{r^{+,s}_{fH} \cdot C3b^{s*} \left(\frac{K^s_{m,I} + fI}{r^s_{cleave}}\right) + K^s_{inhib} + fI^f}. \tag{S28}$$

We estimate the smallest possible concentration of free Factor H molecules fH^s_{free} by providing the maximum number of binding partners ($C3b^{s*} = B_{C3b,max} = 2.01 \cdot 10^{-4}$) to the free Factor H molecules. With the parameters from the **Supplementary Tables S8 - S11** from **Supplementary Section 2.1** the majority of the surface bound molecules are free Factor H molecules:

$$\frac{fH^s_{free}}{fH^s} = 0.99. \tag{S29}$$

Thus, we retrospectively add the same approximation as in the fluid phase in **Supplementary Equation S22**:

$$fH^s = fH^s_{free} + C3b^sH + C3b^sH, I \approx fH^s_{free} \tag{S30}$$

and the steady state of $C3b^sH^*$ simplifies to

$$C3b^sH^* = \frac{r^{+,s}_{fH} \cdot \frac{K^s_{m,I}}{r^s_{cleave}} \cdot fH^s \cdot C3b^{s*}}{K^s_{inhib} + fI^f}. \tag{S31}$$

In order to model the dynamics of the system we do a quasi-steady-state approximation (QSSA) of the intermediate products $C3b^{f/s}H$ and $C3b^{f/s}H, I$ by assuming that these dynamics reach an equilibrium much faster than the dynamics of $C3b^s$. The dynamics of **Supplementary Equation S15** and **S18** are thus simplified to

$$\begin{aligned}\frac{d}{dt}C3b^{f/s} &= -r_{fH}^{+,f/s} \cdot fH^{f/s} \cdot C3b^{f/s} \cdot \frac{fI^f}{K_{inhib}^f/s + fI^f} \\ &= -r_{inhib}^{f/s} \cdot C3b^{f/s},\end{aligned} \tag{S32}$$

$$\begin{aligned}\frac{d}{dt}iC3b^{f/s} &= r_{fH}^{+,f/s} \cdot fH^{f/s} \cdot C3b^{f/s} \cdot \frac{fI^f}{K_{inhib}^{f/s} + fI^f} \\ &= r_{inhib}^{f/s} \cdot C3b^{f/s},\end{aligned} \tag{S33}$$

$$K_{inhib}^{f/s} = \frac{K_{m,I}^{f/s} \cdot r_{fH}^{-,f/s}}{r_{cleave}^{f/s}}. \tag{S34}$$

The inhibition of $C3b$ molecules is directly proportional to the concentration of the $C3b$ molecules with the proportionality constant $r_{inhib}^{f/s}$. The QSSA of the $C3b^{f/s}H, I$ concentration is used in the literature in experimental studies [22], where the kinetics of the regulatory proteins Factor H and Factor I are analyzed. The verification of this QSSA is not possible since the on- and off-rates of the reactions are unknown. Only values for the parameters of the Michaelis-Menten approximation exist [22, 16, 23].

In order to verify the QSSA of $C3b^{f/s}H$, we test conditions that were proposed by Segel and Slemrod [24]. First we will calculate the time scale t_c, the pre steady state period of the fast dynamics that undergo the QSSA. This time scale will be compared to the time scale of the complete system t_s that can be estimated by the maximum change of the system's quantity using

$$t_s = \frac{(C3b^s_{max} - C3b^s_{min})}{\left|\frac{dC3b^s}{dt}\right|_{max}}. \tag{S35}$$

If the time scale of the pre steady state solution is much smaller than the time scale of the system, *i.e.*

$$t_c << t_s, \tag{S36}$$

the QSSA is a valid assumption. Additionally, we calculate the change of the $C3b^s$ concentration during the time period t_c. This change should be negligibly small:

$$\left|\frac{\Delta C3b^s}{C3b^s_0}\right| \approx \frac{1}{C3b^s_0} \cdot \left|\frac{dC3b^s}{dt}\right|_{max} \cdot t_c << 1. \tag{S37}$$

In this section, we will only calculate t_c of the regulation process. The comparison with the system's time scale is done in the results in **Supplementary Section 3.1.2** in **Supplementary Figure S9**. In order to

determine t_c, we determine the dynamics of $C3bH^{f/s}$ with a constant $C3b^{f/s}$ concentration:

$$\frac{d}{dt}C3bH^{f/s} = -\left(r_{fH}^{-,f/s} + \frac{r_{cleave}^{f/s} \cdot fI^f}{K_{m,I}^{f,s}}\right) \cdot C3bH + r_{fH}^{+,f/s} \cdot fH^{f/s} \cdot C3b^{f/s}. \tag{S38}$$

The analytical solution of this ODE is given by

$$C3bH^{f/s}(t) = \frac{r_{fH}^{+,f/s} \cdot \frac{K_{m,I}^{f/s}}{r_{cleave}^{f/s}} \cdot fH^{f/s} \cdot C3b^{f/s*}}{K_{inhib}^{f/s} + fI^f}\left[1 - e^{-\left(r_{fH}^{-,f/s} + \frac{r_{cleave}^{f/s} \cdot fI^f}{K_{m,I}^{f,s}}\right)t}\right] \tag{S39}$$

and we estimate the timescale t_{C3bH} of the process to be

$$t_{C3bH} = \frac{1}{\left(r_{fH}^{-,f/s} + \frac{r_{cleave}^{f/s} \cdot fI^f}{K_{m,I}^{f,s}}\right)} \approx 0.2\,s. \tag{S40}$$

1.5 Amplification of surface-bound $C3b$ molecules

C3b molecules on the cell surface are able to form the C3-convertase enzyme. The $C3b^s$ molecule reacts with a Factor B molecule to C3bB [7, 8]. The Factor B molecule is cleaved by Factor D and the C3-convertase C3bBb is formed [25]. Factor H interfers sterically with C3-convertase molecules and

Table S6. Biochemical reactions of the formation of surface bound C3-convertase

(1)	$C3b^s + fB^f \xrightarrow{r_{C3bB}^{+}} C3b^sB$	Generation of $C3b^sB$ [7]
(2)	$C3b^sB \xrightarrow{r_{C3bB}^{+}} C3b^s + fB^f$	Sponateous release of Factor B [7]
(3)	$C3b^sB \xrightarrow{r_{C3bBb,cat},\, fD^f} C3b^sBb$	Enzymatical cleavage of Factor B by Factor D [7]
(4)	$C3b^sBb + fP^f \xrightarrow{r_{C3bBbP,fP}^{+}} C3b^sBbP$	Stabilization of $C3b^sBb$ with Factor P [7]
(5)	$C3b^sBbP \xrightarrow{r_{C3bBbP}^{-}} C3b^sBb + fP^f$	Sponateous release of Factor P [7]
(6)	$C3b^sBb \xrightarrow{r_{C3bBb}^{-}} C3b^s + Bb^f$	Spontaneous decay of $C3b^sBb$ [7]
(7)	$C3b^sBb \xrightarrow{r_{C3bBb,fH}^{-} \cdot fH^s} C3b^s + Bb^f$	Factor H assisted decay of $C3b^sBb$ [26, 27]
(8)	$C3b^sBbP \xrightarrow{r_{C3bBbP}^{-}} C3b^s$	Spontaneous decay of $C3b^sBbP$ [7]
(9)	$C3b^sBbP \xrightarrow{r_{C3bBbP,fH}^{-} \cdot fH^s} C3b^s + Bb^f$	Factor H assisted decay of $C3b^sBb$ [26, 27]

dissociates the Bb fragment from the C3-convertase molecule[10]. The molecule $C3b^sB$ is not affected

by Factor H, while the molecule $C3b^sBb$ and $C3b^sBbP$ are [26]. The molecule properdin (fP^f) acts as a stabilizer for C3b products. It increases the lifetime of the C3b products and slows down the Factor H-induced decay of the products [28, 27]. Properdin binds to C3b with higher polyvalent avidity for clustered surface-bound C3b, *i.e.* a higher affinity for $C3bBb > C3bB > C3b$ complexes [29, 9, 30]. Thus, reactions, in which properdin associates to smaller complexes, are neglected for the sake of a simple model. The considered biochemical reactions are summarized in **Supplementary Table S6** and are visualized in the green box of **Supplementary Figure S1**.

The following ODEs are derived from the reactions in **Table S6** using the law of mass action:

$$\begin{aligned}\frac{d}{dt}C3b^s_{amplification} &= -r^+_{C3bB}\cdot fB^f\cdot C3b^s + r^-_{C3bB}\cdot C3b^sB\\ &+\left(r^-_{C3bBb} + r^-_{C3bBb,fH}\cdot fH^s\right)C3b^sBb\\ &+\left(r^-_{C3bBbP} + r^-_{C3bBbP,fH}\cdot fH^s\right)\cdot C3b^sBbP,\end{aligned} \tag{S41}$$

$$\frac{d}{dt}C3b^sB = r^+_{C3bB}\cdot fB^f\cdot C3b^s - \left(\frac{r_{C3bBb,cat}\cdot fD^f}{K_{cat,fD}+fD^f} + r^-_{C3bB}\right)\cdot C3b^sB, \tag{S42}$$

$$\frac{d}{dt}C3b^sBb = \frac{r_{C3bBb,cat}\cdot fD^f}{K_{cat,fD}+fD^f}\cdot C3b^sB - \left(r^+_{C3bBbP,fP}\cdot fP^f + r^-_{C3bBb} + r^-_{C3bBb,fH}\cdot fH^s\right)\cdot C3b^sBb, \tag{S43}$$

$$\frac{d}{dt}C3b^sBbP = r^+_{C3bBbP,fP}\cdot fP^f\cdot C3b^sBb - \left(r^-_{C3bBbP} + r^-_{C3bBbP,fH}\cdot fH^s\right)\cdot C3b^sBbP. \tag{S44}$$

The term $\frac{d}{dt}C3b^s_{amplification}$ describes the change of the $C3b^s$ concentration with time due to amplification dynamics. From **Supplementary Equations S42 - S44** we can calculate the steady states of the intermediate products of the C3-convertase at the cell surface

$$C3b^sB^* = \frac{r^+_{C3bB}\cdot fB^f}{\left(\frac{r_{C3bBb,cat}\cdot fD^f}{K_{cat,fD}+fD^f} + r^-_{C3bB}\right)}\cdot C3b^{s*}, \tag{S45}$$

$$C3b^sBb^* = \frac{\frac{r_{C3bBb,cat}\cdot fD^f}{K_{cat,fD}+fD^f}}{\left(r^+_{C3bBbP,fP}\cdot fP^f + r^-_{C3bBb} + r^-_{C3bBb,fH}\cdot fH^s\right)}\cdot C3b^sB^*, \tag{S46}$$

$$C3b^sBbP^* = \frac{r^+_{C3bBbP,fP}\cdot fP^f}{\left(r^-_{C3bBbP} + r^-_{C3bBbP,fH}\cdot fH^s\right)}\cdot C3b^sBb^*. \tag{S47}$$

We introduce the following parameters for a shorter representation of the terms:

$$k_{C3bB} = \frac{r^{+}_{C3bB} \cdot fB^{f}}{\left(\frac{r_{C3bBb,cat} \cdot fD^{f}}{K_{cat,fD}+fD^{f}} + r^{-}_{C3bB}\right)}, \tag{S48}$$

$$k_{C3bBb} = \frac{\frac{r_{C3bBb,cat} \cdot fD^{f}}{K_{cat,fD}+fD^{f}}}{\left(r^{+}_{C3bBbP,fP} \cdot fP^{f} + r^{-}_{C3bBb} + r^{-}_{C3bBb,fH} \cdot fH^{s}\right)}, \tag{S49}$$

$$k_{C3bBbP} = \frac{r^{+}_{C3bBbP,fP} \cdot fP^{f}}{\left(r^{-}_{C3bBbP} + r^{-}_{C3bBbP,fH} \cdot fH^{s}\right)}, \tag{S50}$$

$$k_{Convertase} = k_{C3bB} + k_{C3bB} \cdot k_{C3bBb} + k_{C3bB} \cdot k_{C3bBb} \cdot k_{C3bBbP}. \tag{S51}$$

If we multiply the steady state concentration $C3b^{s*}$ with $k_{Convertase}$ we get the concentration of occupied binding sites by the intermediate products of C3-convertase.

The cleavage of new $C3b^{f}$ molecules by the surface bound C3-convertase molecules is done by the chemical reactions in **Supplementary Table S7**.

Table S7. Amplification of the alternative pathway

(9a)	$C3^{f} + C3b^{s}Bb \underset{r^{-}_{C3b^{s}Bb,C3}}{\overset{r^{+}_{C3b^{s}Bb,C3}}{\rightleftarrows}} C3^{f}, C3b^{s}Bb$
(9b)	$C3^{f} + C3b^{s}BbP \underset{r^{-}_{C3b^{s}Bb,C3P}}{\overset{r^{+}_{C3b^{s}BbP,C3}}{\rightleftarrows}} C3^{f}, C3b^{s}BbP$
(10a)	$C3^{f}, C3b^{s}Bb \xrightarrow{r_{amp,cleave}} C3b^{f} + C3b^{s}Bb$
(10b)	$C3^{f}, C3b^{s}BbP \xrightarrow{r_{amp,cleave\_stab}} C3b^{f} + C3b^{s}BbP$

From these reactions we derive the formation of activated $C3b^{f}$ by amplification of the alternative pathway using Michaelis-Menten kinetics:

$$\frac{d}{dt}C3b^{f}_{amplification} = r_{amp,cleave}\frac{C3b^{s}Bb \cdot C3^{f}}{K_{m,amp} + C3^{f}} + r_{cleave\_stab}\frac{C3b^{s}BbP \cdot C3^{f}}{K_{m,amp\_stab} + C3^{f}}. \tag{S52}$$

Using the C3-convertase concentrations from **Supplementary Equations S46** and **S47** and assuming that properdin does not affect the enzymatic properties of the convertase complex ($r_{cleave} = r_{cleave\_stab}$

and $K_{m,amp} = K_{m,amp\_stab}$), we combine both cleavage reactions:

$$\frac{d}{dt}C3b^f_{amplification} = r_{amp,cleave} \cdot (C3b^sBb^* + C3b^sBbP^*) \cdot \frac{C3^f}{K_{m,amp} + C3^f}, \tag{S53}$$

$$\begin{aligned}\frac{d}{dt}C3b^f_{amplification} &= r_{amp,cleave} \cdot k_{C3bB} \cdot k_{C3bBb}\left(1 + k_{C3bBbP}\right) \cdot \frac{C3^f}{K_{m,amp} + C3^f} \cdot C3b^s \\ &= r_{amp}(fH^s) \cdot C3b^s.\end{aligned} \tag{S54}$$

The inflow is directly proportional to the surface bound $C3b^s$ concentration with the proportionality constant $r_{amp}(fH^s)$. The effective rate $r_{amp}(fH^s)$ depends on the concentration of surface bound Factor H (fH^s) and decreases with increasing fH^s.

We verify the QSSA using the same approach as in **Supplementary Section 1.4** as proposed by Segel and Slemrod. First we calculate the analytical solution of the dynamics of the C3-convertase molecules under the assumption of constant $C3b^s$ molecules. The resulting dynamics are:

$$C3b^sB(t) = k_{C3bB} \cdot C3b^s \left[1 - e^{-\left(r^+_{C3bB} \cdot fB^f + r^-_{C3bB}\right)t}\right], \tag{S55}$$

$$\begin{aligned}C3b^sBb(t) &= k_{C3bB} \cdot k_{C3bBb} \cdot C3b^s \left[1 - e^{-\left(r^+_{C3bBbP,fP} \cdot fP^f + r^-_{C3bBb} + r^-_{C3bBb,fH} \cdot fH^s\right)t}\right] \\ &+ k_{C3bB} \frac{\frac{r_{C3bBb,cat} \cdot fD^l}{K_{cat,fD} + fD^f} \cdot \left[e^{-\left(r^+_{C3bBbP,fP} \cdot fP^f + r^-_{C3bBb} + r^-_{C3bBb,fH} \cdot fH^s\right)t} - e^{-\left(\frac{r_{C3bBb,cat} \cdot fD^l}{K_{cat,fD} + fD^f} + r^-_{C3bB}\right)t}\right]}{\left[\left(r^+_{C3bBbP,fP} \cdot fP^f + r^-_{C3bBb} + r^-_{C3bBb,fH} \cdot fH^s\right) - \left(\frac{r_{C3bBb,cat} \cdot fD^l}{K_{cat,fD} + fD^f} + r^-_{C3bB}\right)\right]},\end{aligned} \tag{S56}$$

$$\begin{aligned}C3b^sBbP(t) &= k_{C3bB} \cdot k_{C3bBb} \cdot k_{C3bBbP} \cdot C3b^s \left[1 - e^{-\left(r^-_{C3bBbP} + r^-_{C3bBbP,fH} \cdot fH^s\right)t}\right] \\ &+ k_{C3bB} \cdot k_{C3bBb} \frac{r^+_{C3bBbP,fP} \cdot fP^f \cdot \left[e^{-\left(r^-_{C3bBbP} + r^-_{C3bBbP,fH} \cdot fH^s\right)t} - e^{-\left(r^+_{C3bBbP,fP} \cdot fP^f + r^-_{C3bBb} + r^-_{C3bBb,fH} \cdot fH^s\right)t}\right]}{\left[r^-_{C3bBbP} + r^-_{C3bBbP,fH} \cdot fH^s - \left(r^+_{C3bBbP,fP} \cdot fP^f + r^-_{C3bBb} + r^-_{C3bBb,fH} \cdot fH^s\right)\right]}.\end{aligned} \tag{S57}$$

It follows that the dynamics have the same steady state as **Supplementary Equations S45 - S47** and their corresponding time scales are:

$$t_{C3b^sB} = \frac{1}{\frac{r_{C3bBb,cat} \cdot fD^l}{K_{cat,fD} + fD^l} + r^-_{C3bB}} \approx 2.5\, s, \tag{S58}$$

$$t_{C3b^sBb} = \frac{1}{r^+_{C3bBbP,fP} \cdot fP^l + r^-_{C3bBb} + r^-_{C3bBb,fH} \cdot fH^s}, \tag{S59}$$

$$t_{C3b^sBbP} = \frac{1}{r^-_{C3bBbP} + r^-_{C3bBbP,fH} \cdot fH^s}. \tag{S60}$$

The timescales of $C3b^sBb$ and $C3b^sBbP$ depend on the fH^s concentration and are given in **Supplementary Figure S2** for the fH^s interval used in this study. According to the literature, the

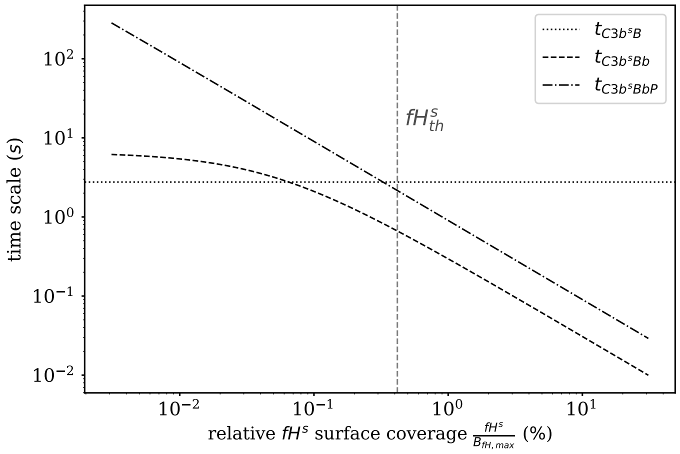

Figure S2. Time scales of the C3-convertase formation for various Factor H concentrations on the cell surface.

formation of the C3-convertase molecules is a fast process [7, 31] and previous models even assumed an instantaneous formation of the molecule [31].

1.6 Spatial distribution of fluid phase $C3b$ molecules

Modeling the interaction of cell surfaces with complement molecules in the fluid phase poses some challenges. Models based on ordinary differential equations are only suitable to a limited extent, since the basic requirement is a well mixed system of the modeled molecules. For the modeled molecules on cell surfaces ($C3b^s$, fH^s, $iC3bs$, ...) this does not apply, since these molecules occur only on cell surfaces, especially on complement activating cell surfaces. Additionally huge concentration differences occur for $C3b^f$ molecules in the vicinity of activating surfaces and the surrounding volume. For this reason, in contrast to existing publications [14, 15, 32, 16, 33], we will choose an approach that takes into account the spatial distribution of the complement molecules.

$C3b^f$ in aqueous solutions has a short half-life time of $60\,\mu s$ [7] and its decay is modeled by an exponential rate law (see **Supplementary Table S4**). We estimate the volume in which activated molecules can diffuse before they are deactivated by calculating the mean square displacement

$$\langle r^2 \rangle = 6 \cdot D_{C3b^f} \cdot t. \tag{S61}$$

Almost all $C3b^f$ are inactivated before their mean square displacement reaches more than $200\,nm$ (Supplementary **Figure S3A**). Thus, the radius of the spherical activation volume is not larger than $200\,nm$. If we map this diffusion distance to $C3b^f$ molecules that are able to reach the cell surface, we can conclude that the volume in which the activated molecules diffuse is only a very thin sphere around the cell

(Supplementary **Figure S3B**). Activating cells with a concentration of 10^{10} cells$/l$, which is clearly above the cell count of typical infection scenarios, have a center-center distance of $46\,\mu m$. The center-center distance of activating cells is much larger than the average distance that an activated $C3b^f$ molecule can diffuse (Supplementary **Figure S3C**). Thus, activated molecules emerging from a cell surface will most likely not interact with neighboring cell surfaces. For this reason, we will only consider a single spherical cell in our model with the coordinates origin in the cell center. To incorporate the effect of these spatial

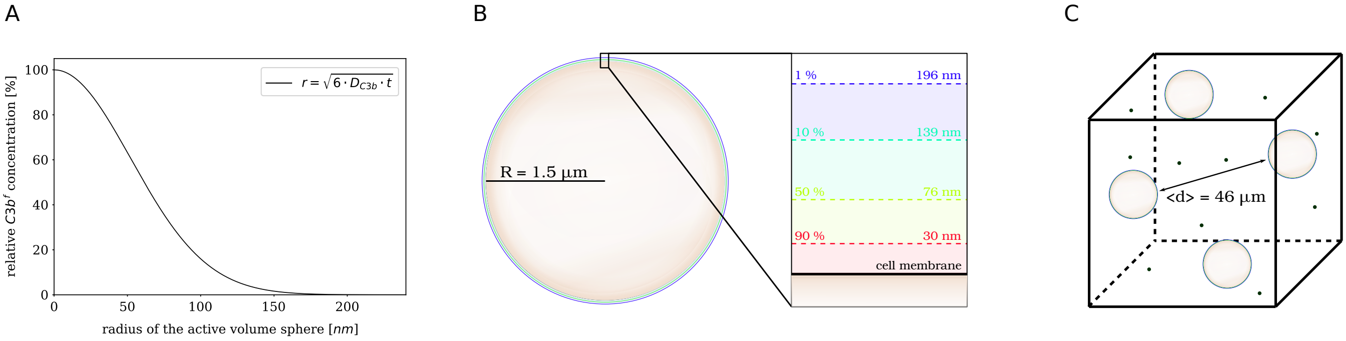

Figure S3. Spatial distribution of $C3b^f$. **(A)** Proportion of active $C3b^f$ molecules as a function of the molecules mean square displacement, before it gets inactivated. Based on the exponential decay of $C3b^f$ molecules and the mean square displacement of the molecules, we estimate the maximal volume of active $C3b^f$ molecules. **(B)** The volume of activated $C3b^f$ emerging at the cell surface is compared to the cell volume only a small sphere around the activating cell. **(C)** The center-center distance of 10^{10} activating cells in a volume of $1\,l$ is about $46\,\mu m$ and is much larger than the region of increased active $C3b^f$ concentration around the activating cell, which is about $0.2\,\mu m$: Molecular exchange between cells is highly unlikely.

inhomogeneities, we model the $C3b^f$ concentration as a function of the distance from the cell center by using diffusion reaction kinetics:

$$\frac{d}{dt}C3b^f(r,t) = D_{C3b}\Delta_r C3b^f - \left(r^f_{inhib} + r^f_{stab}\right) \cdot C3b^f + A, \qquad R < r < \infty. \tag{S62}$$

For reasons of radial symmetry, the concentration profile depends only on the distance to the cell center r. The $C3b^f$ concentration is only defined for distances larger than the cell radius R. The diffusion coefficient D_{C3b} was calculated, using Stokes–Einstein equation, for the diffusion of spherical particles. The inactivation processes (r^f_{inhib} + r_{stab}) as well as the spontaneous activation (A) can take place in the entire space around the cell. The boundary condition at the cell surface ($r = R$) represents Fick's first law:

$$\left.\frac{\partial C3b^f(r)}{\partial r}\right|_{r=R} = -\frac{j}{D_{C3b}}. \tag{S63}$$

The flow of molecules j at the surfaces is the net change of molecules that attach to the surface (opsonization) and that arise at the surface due to amplification reactions. The flow depends on the molecule concentrations on the cell surface that are given by the well-mixed system of surface-bound molecules. The well-mixed fluid system without any cells is described by **Supplementary Equation S62** without the diffusion term $\Delta_r C3b^f$. We use the steady state of the well-mixed system to define our second boundary conditions for

large distances to the cell center

$$C3b^f(r \to \infty) = \frac{A}{r^f_{inhib} + r_{stab}}. \tag{S64}$$

The derivation of the concentration profile of the fluid phase $C3b^f$ molecule is taken from Heinrich *et al.* [34]. They derive a time-dependent and a steady state solution of a complement concentration around a spherical cell for a system with a constant source. In contrast to Heinrich *et al.* [34] our model has no constant source but is coupled to the molecule concentrations at the cell surface. These molecules influence the flow of $C3b$ molecules at the cell surface. On the one hand, $C3b$ molecules bind covalently to the surface and, on the other hand, $C3b$ molecules are created on the cell surface by the amplification process. The boundary condition **S63** is thus described by the following flow of C3b molecules:

$$\left.\frac{\partial C3b^f(r)}{\partial r}\right|_{r=R} = -\frac{j}{D_{C3b}} = -\frac{r_{amp}(fH^s) \cdot C3b^s - r_{ops} \cdot B_{C3b,free} \cdot C3b^f(R)^*}{D_{C3b}}. \tag{S65}$$

The steady state solution of **Supplementary Equation S62** is given by

$$C3b^f(r)^* = \frac{\left(r_{amp}(fH^s) \cdot C3b^s - r_{ops} \cdot B_{C3b,free} \cdot C3b^f(R)^*\right) \cdot R^2}{D_{C3b} + R\sqrt{D_{C3b}\left(r^f_{inhib} + r_{stab}\right)}} \frac{e^{\left(-\frac{r-R}{\sqrt{D_{C3b}/\left(r^f_{inhib}+r_{stab}\right)}}\right)}}{r} + \frac{A}{\left(r^f_{inhib} + r_{stab}\right)}. \tag{S66}$$

This results in a $C3b^f$ concentration at the cell surface

$$C3b^f(R)^* = \frac{r_{amp}(fH^s) \cdot C3b^s \cdot R + \frac{A}{\left(r^f_{inhib}+r_{stab}\right)}\left(R\sqrt{D_{C3b}\left(r^f_{inhib} + r_{stab}\right)} + D_{C3b}\right)}{r_{ops} \cdot B_{C3b,free} \cdot R + R\sqrt{D_{C3b}\left(r^f_{inhib} + r_{stab}\right)} + D_{C3b}}. \tag{S67}$$

The first terms in the numerator, $r_{amp}(fH^s) \cdot C3b^s \cdot R$, and denominator, $r_{ops} \cdot B_{C3b,free} \cdot R$, of the solution depend on cell surface related processes. The first term of the numerator is related to the amplification process and is for small fH^s concentrations below $10\ \frac{molecule}{\mu m^2}$ up to five orders of magnitude larger than the second term of the numerator, which is related to fluid phase reactions like the spontaneous activation and diffusion (see **Supplementary Figure S4A**). The first term decreases with increasing fH^s concentrations. The first term of the denominator, $r_{ops} \cdot B_{C3b,free} \cdot R$, only contributes to the total denominator at high fH^s concentrations above $10^2\ \frac{molecule}{\mu m^2}$. This opsonization related term is responsible for the fact that the $C3b^f(R)$ drops below the serums equilibrium concentration at high fH^s concentrations.

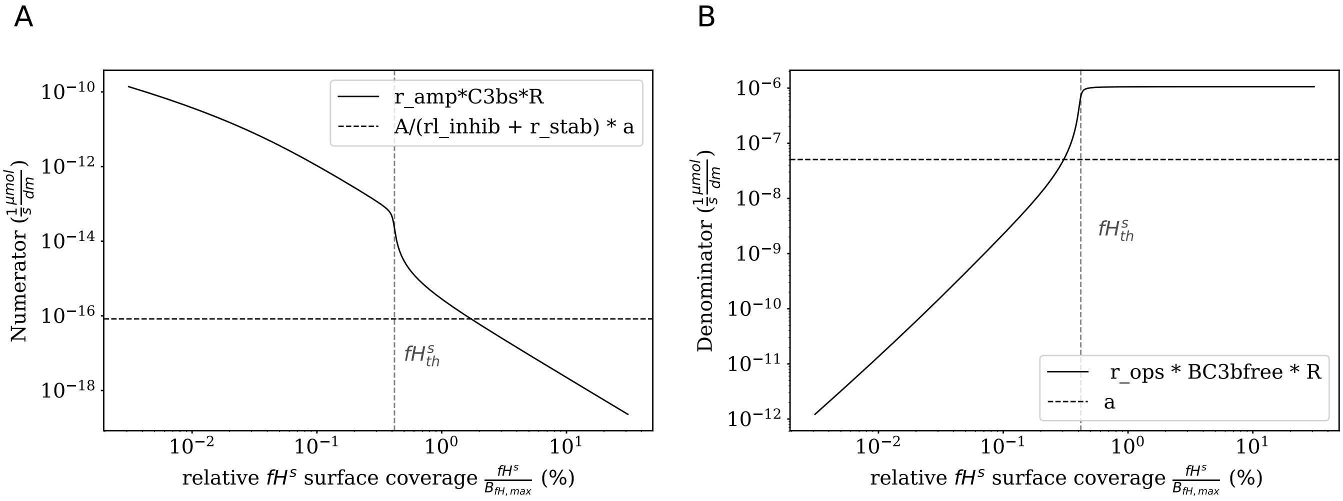

Figure S4. $C3b^f$ concentration at the cell surace. Terms of the steady state solution of $C3b^f(R)$ at the cell surface as a function of fH^s ; **(A)** Terms of the numerator. **(B)** Terms of the denominator.

The dynamics of the surface bound $C3b$ molecules are coupled to the fluid-phase $C3b$ molecule concentration at the cell via the opsonization process (compare Supplementary **Supplementary Equation S13**). For the dynamics on the cell surface it is therefore sufficient to consider only the concentration of $C3b^f$ at the cell surface $C3b^f(R)$. For this concentration we also make a QSSA. Given a constant $C3b^s$ concentration the analytical solution of **Supplementary Equation S62** is

$$
\begin{aligned}
&C3b^f(r,t) = \\
&\frac{j\cdot R^2}{r}\left\{\begin{array}{l}
\frac{1}{2\left(D_{C3b}+R\sqrt{D_{C3b}\left(r^f_{inhib}+r_{stab}\right)}\right)}\exp\left(-\frac{r-R}{\sqrt{D_{C3b}/\left(r^f_{inhib}+r_{stab}\right)}}\right)\mathrm{erfc}\left(\frac{r-R}{2\sqrt{D_{C3b}t}}-\sqrt{\left(r^f_{inhib}+r_{stab}\right)t}\right)\\
+\frac{1}{2\left(D_{C3b}-R\sqrt{D_{C3b}\left(r^f_{inhib}+r_{stab}\right)}\right)}\exp\left(\frac{r-R}{\sqrt{D_{C3b}/\left(r^f_{inhib}+r_{stab}\right)}}\right)\mathrm{erfc}\left(\frac{r-R}{2\sqrt{D_{C3b}t}}+\sqrt{\left(r^f_{inhib}+r_{stab}\right)t}\right)\\
-\frac{1}{D_{C3b}-\left(r^f_{inhib}+r_{stab}\right)R^2}\exp\left(\frac{r-R}{R}+\left(\frac{D_{C3b}}{R^2}-\left(r^f_{inhib}+r_{stab}\right)\right)t\right)\mathrm{erfc}\left(\frac{r-R}{2\sqrt{D_{C3b}t}}+\frac{\sqrt{D_{C3b}t}}{R}\right)
\end{array}\right\}\\
&+\frac{A}{\left(r^f_{inhib}+r_{stab}\right)}.
\end{aligned}
\tag{S68}
$$

The time scale of the concentration process depends on the diffusion constant D_{C3b} and on the decay rates r^f_{inhib} and r_{stab} and is not related to the flow of molecules j at the cell surface. **Supplementary Figure S5** shows the concentration profile of $C3b^f(R)$ molecules at the cell surface for a constant inflow j. The time-dependent solution equilibrates within $t_{C3b^f(R)} = 10^{-3}\,s$ into the steady state solution at the cell surface with the given rates, which is approximately two orders of magnitude smaller than the timescales of the intermediate product $C3bH$, where we also perform a QSSA.

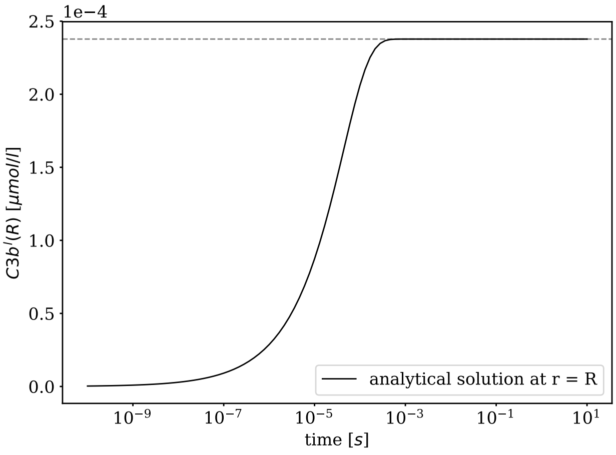

Figure S5. Dynamics of the $C3b^f$ concentration at the cell surface ($R = 1.5\,\mu m$). The steady state is reached within $10^{-3}\,s$.

Analogous to the dynamics of the $C3b^f(r)$ molecules we model the dynamics of the anaphylatoxin $C3a^f(r)$:

$$\begin{aligned} &\frac{d}{dt}C3a^f(r,t) = D_{C3a}\Delta_r C3a^f - r_{dec,C3a}\cdot C3a^f + A, \\ &\qquad\qquad R < r < \infty. \end{aligned} \tag{S69}$$

The spontaneous activation A is identical to the activation of the $C3b^f$ molecules, since the cleavage of one $C3$ molecule results in one $C3a$ and one $C3b$ molecule. The boundary conditions are given by

$$\left.\frac{\partial C3a^f(r)}{\partial r}\right|_{r=R} = -\frac{j}{D_{C3a}} = -\frac{r_{amp}(fH^s)\cdot C3b^s}{D_{C3a}} \tag{S70}$$

and

$$C3a^f(r\to\infty) = \frac{A}{r_{dec,C3a}}. \tag{S71}$$

The boundary condition at the cell surface contains, in contrast to **Supplementary Equation S63**, only the outflow from the surface, as no $C3a$ molecules bind to the surface. The steady state solution of **Supplementary Equation S69** is given by

$$C3a^f(r)^* = \frac{r_{amp}(fH^s)\cdot C3b^s\cdot R^2}{D_{C3b} + R\sqrt{D_{C3a}\left(r_{dec,C3a}\right)}}\frac{e^{\left(-\frac{r-R}{\sqrt{D_{C3b}/r_{dec,C3a}}}\right)}}{r} + \frac{A}{r_{dec,C3a}}. \tag{S72}$$

2 PARAMETERS OF THE DYNACOSYS MODEL

2.1 Model parameters

Table S8. Complement protein concentrations

Molecule	Concentration $\left(\frac{\mu mol}{l}\right)$	Source
$C3^f$	6.49	[35]
fH^f	3.61	[35]
fI^f	0.46	[35]
fB^f	2.15	[35]
fD^f	0.13	[35]
fP^f	0.38	[35]

Table S9. Binding site concentrations

Molecule	Concentration $\left(\frac{\mu mol}{dm^2}\right)$	Source
$B^s_{C3b,max}$	$2.01 \cdot 10^{-4}$	calculated from [5, 20, 21]
$B^s_{fH,max}$	$9.23 \cdot 10^{-4}$	calculated from [4]
$B^s_{fH,min}$	$1.02 \cdot 10^{-6}$	calculated from [1]

Table S10. Reactions in the fluid

Chemical reaction	Rate constant	Value	Source
diffusion of $C3b^f$	D_{C3b}	$1.0 \cdot 10^{-9}\ \frac{dm^2}{s}$	calculated from [21]
diffusion of $C3a^f$	D_{C3a}	$1.9 \cdot 10^{-9}\ \frac{dm^2}{s}$	calculated from [36]
spontaneous activation of $C3b^f$ molecules	A	$1.8 \cdot 10^{-5}\ \frac{1}{s}\frac{\mu mol}{l}$	calculated from [17, 37]
spontaneous inactivation of $C3b^f$ thioester bond	r_{stab}	$1.1 \cdot 10^{4}\ \frac{1}{s}\frac{\mu mol}{l}$	calculated from [7]
decay of $C3a^f$	r_{decC3a}	$5.7 \cdot 10^{-3}\ \frac{1}{s}$	[38]
association of FH^f to $C3b^f$	r_{fH}^{+f}	$5.2\ \frac{1}{s}\frac{l}{\mu mol}$	[37, 22, 7]
dissociation of complex $C3b^f H$	r_{fH}^{-f}	$3.25\ \frac{1}{s}$	[22]
cleavage of $C3b^f HI$ complex	r_{cleave}^{f}	$1.32\ \frac{1}{s}$	[22]
Michaelis-Menten constant of cleavage of $C3b^f HI$ complex	K_{mI}^{f}	$0.252\ \frac{\mu mol}{l}$	[22]

Table S11. Reactions on the surface

Chemical reaction	Rate constant	Value	Source
dissociation constant of FH^s to surface	K_{dfH}	$9\,\frac{\mu mol}{l}$	[13]
association of $C3b$ to the surface	r_{ops}	$3.6 \cdot 10^2\,\frac{1}{s}\frac{l}{\mu mol}$	[31, 19]
association of FH^s to $C3b^s$	r_{fH}^{+s}	$1 \cdot 10^2\,\frac{1}{s}\frac{dm^2}{\mu mol}$	estimated based on [39, 29, 7, 40]
dissociation of complex $C3b^sH$	r_{fH}^{-s}	$3.25\,\frac{1}{s}$	analogous to [22]
cleavage of $C3b^fHI$ complex	r_{cleave}^{s}	$1.32\,\frac{1}{s}$	analogous to [22]
Michaelis-Menten constant of cleavage of $C3b^sHI$ complex	K_{mI}^{s}	$0.252\,\frac{\mu mol}{l}$	analogous to [22]
association of fB^f to $C3b^s$	r_{C3bB}^{+}	$1.36 \cdot 10^{-4}\,\frac{1}{s}\frac{l}{\mu mol}$	[41, 42]
dissociation of $C3b^sB$	r_{C3bB}^{-}	$1.15 \cdot 10^{-1}\,\frac{1}{s}$	[41, 42]
cleavage of $C3b^sB$ complex	r_{catfD}	$5.0\,\frac{1}{s}$	[43]
Michaelis-Menten constant of cleavage of $C3b^sB$ complex	K_{catfD}	$2.5\,\frac{\mu mol}{l}$	[43]
association of fP^f to $C3b^sBb$	r_{C3bBbP}^{+}	$0.4\,\frac{1}{s}\frac{l}{\mu mol}$	[9, 30]
dissociation of $C3b^sBb$	r_{C3bBb}^{-}	$1.4 \cdot 10^{-4}\,\frac{1}{s}$	[27]
dissociation of $C3b^sBbP$	r_{C3bBb}^{-}	$8.3 \cdot 10^{-5}\,\frac{1}{s}$	[27]
Factor H driven dissociation of $C3b^sBb$	$r_{C3bBb,fH}^{-}$	$3.5 \cdot 10^5\,\frac{1}{s}\frac{l}{\mu mol}$	estimated from [27]
Factor H driven dissociation of $C3b^sBbP$	$r_{C3bBb,fH}^{-}$	$1.2 \cdot 10^5\,\frac{1}{s}\frac{l}{\mu mol}$	estimated from [27]
cleavage of $C3$ by C3-convertase	$r_{amp,cleave}$	$1.78\,\frac{1}{s}$	[7, 8]
Michaelis-Menten constant of cleavage of $C3$ by C3-convertase	$K_{m,amp}$	$5.9\,\frac{\mu mol}{l}$	[7, 8]
decay of surface bound $iC3b^s$	r_{dec}	$2.4 \cdot 10^{-5}\,\frac{1}{s}$	[31, 44]

The rates r_{amp} and r^s_{inhib} that are respectively defined in **Equation S54** and **Equation S32** depend on fH^s and will be variied in the course of our analysis. Both rates are shown in the **Supplementary Figure S6**. We define the fH^s concentration, at which r_{amp} and r^s_{inhib} balance out as fH^s_{th}:

$$r_{amp}(fH^s_{th}) = r^s_{inhib}(fH^s_{th}). \tag{S73}$$

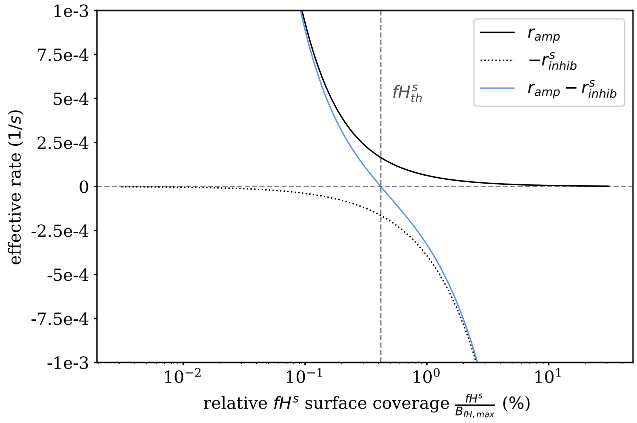

Figure S6. Effective rates $r_{amp}(fH^s)$ and $r^s_{inhib}(fH^s)$ over the fH^s concentration. The threshold fH^s_{th} is defined by $r_{amp}(fH^s_{th}) = r^s_{inhib}(fH^s_{th})$

3 ANALYSIS OF THE DYNACOSYS MODEL

3.1 Complement dynamics of C3b-opsonization requires hybrid differential equation approach

3.1.1 Steady state solution

The dynamics of surface bound $C3b^s$ and $iC3b^s$ concentrations are obtained by combining the **Supplementary Sections 1.1 - 1.6**. The resulting ODEs are given by

$$\frac{d}{dt}C3b^s = r_{ops} \cdot B_{C3b,free}(C3b^s, iC3b^s) \cdot C3b^f(R)^* - r^s_{inhib}(fH^s) \cdot C3b^s, \tag{S74}$$

$$\frac{d}{dt}iC3b^s = r^s_{inhib}(fH^s) \cdot C3b^s - r_{dec} \cdot iC3b^s. \tag{S75}$$

The **Supplementary Equations S74** and **S75** include the QSSAs from **Supplementary Sections 1.1 - 1.6**. We derive the steady states of the surface-bound molecules using the following substitutions to present the whole solution in a concise manner:

$$a = R\sqrt{D_{C3b}\left(r^f_{inhib} + r_{stab}\right)} + D_{C3b}, \tag{S76}$$

$$b = 1 + k_{Convertase} + \frac{r^s_{inhib}}{r_{dec}}, \tag{S77}$$

$$c = r^s_{inhib}(fH^s) - r_{amp}(fH^s). \tag{S78}$$

The substitution a combines parameters of the fluid phase whereas substitution b describes the ratio of surface-bound molecules. The parameters $k_{Convertase}$ and $\frac{r^s_{inhib}}{r_{dec}}$ multiplied by the $C3b^s$ concentration give the concentrations of C3-convertase molecules and $iC3b^s$ molecules, respectively. The solution of the steady state of $C3b^s$ molecules corresponds to the solution of a quadratic equation $C3b^{s*} = \left(p \pm \sqrt{p^2 - 4qN}\right)/2N$ and thus contains two solution branches.

$$C3b^{s*} = \frac{\left(R \cdot B_{C3b,max} \cdot r_{ops} \cdot c + \frac{a \cdot b \cdot r_{ops} \cdot A}{\left(r^f_{inhib} + r_{stab}\right)} + a \cdot r^s_{inhib}\right)}{2 \cdot r_{ops} \cdot R \cdot b \cdot c}$$
$$\pm \frac{\sqrt{\left(R \cdot B_{C3b,max} \cdot r_{ops} \cdot c + \frac{a \cdot b \cdot r_{ops} \cdot A}{\left(r^f_{inhib} + r_{stab}\right)} + a \cdot r^s_{inhib}\right)^2 - 4\frac{a \cdot r_{ops} \cdot B_{C3b,max} \cdot A}{\left(r^f_{inhib} + r_{stab}\right)} \cdot r_{ops} \cdot R \cdot b \cdot c}}{2 \cdot r_{ops} \cdot R \cdot b \cdot c} \tag{S79}$$

The steady state concentration of the inactive $iC3b^s$ molecules is related to the active $C3b^s$ molecules by the following equation:

$$iC3b^{s*} = \frac{r^s_{inhib}}{r_{dec}} C3b^{s*}. \tag{S80}$$

The effective rate r^s_{inhib}, which is directly proportional to the concentration of Factor H molecules on the cell surface, increases the $iC3b^s$ concentration. A smaller decay rate r_{dec} increases the $iC3b^s$ concentration.

The variation of the fH^s concentration shows that singularities occur if $c = r^s_{inhib}(fH^s_{th}) - r_{amp}(fH^s_{th}) = 0$. Out of in total three singularities only one singularity has a positive value ($fH^s_{th} =$

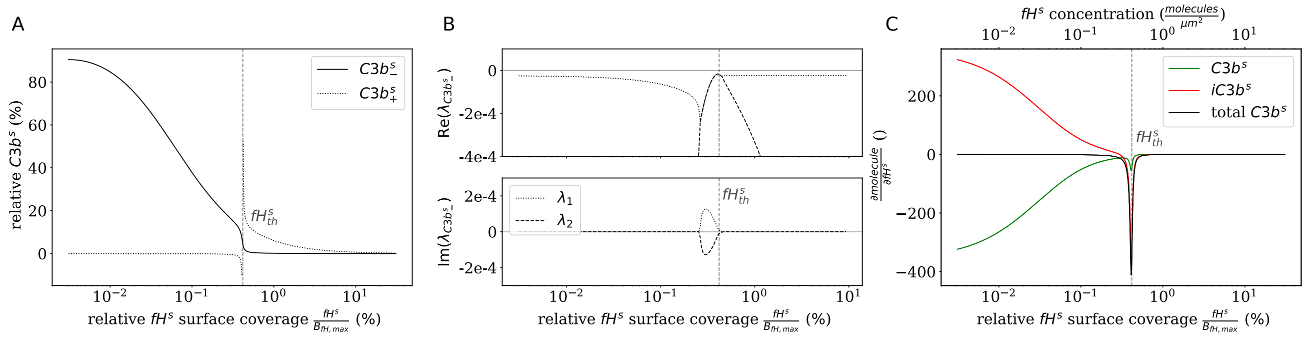

Figure S7. Steady state results of $C3b^s$ molecules for varying surface bound Factor H concentrations. **(A)** Both branches of the $C3b^s$ steady state solution: The solid line indicates the continuous biological solution, while the dashed line shows the discontinuous solution. The pole of the discontinuous solution occurs at fH^s_{th}. **(B)** Stability analysis of the continuous biological solution: The biological solution is stable for all tested parameter sets. **(C)** Derivative of molecule concentrations with respect to the fH^s concentration. fH^s_{th} marks an extremum of the $C3b^s(fH^s)$ function representing the steepest change of molecule concentration in the transition region with respect to Factor H.

230 $\frac{molecules}{\mu m^2}$). While the positive branch of the steady state solution contains an infinite discontinuity, the negative branch of the steady state solution contains a removable singularity and is the only biological solution (**Supplementary Figure S7A**). The negative branch is the only stable steady state over the complete range of fH^s concentrations (**Supplementary Figure S7B**). At the singularity fH^s_{th} the derivatives of the molecule concentrations with respect to fH^s show an extremum (**Supplementary Figure S7C**). There is even a global extremum for the total concentration of all C3b molecules on the surface.

3.1.2 Dynamics

Before we solve the dynamics of **Supplementary Equations S74** and **S75** numerically, we have to check, if the QSSAs of **Supplementary Sections 1.4 - 1.6** are valid. We compare the timescale t_{C3b^s} with the timescales of the QSSAs. We will compute t_{C3b^s} using Segels and Slemrods approach:

$$t_{C3b^s} = \frac{(C3b^s_{max} - C3b^s_{min})}{\left|\frac{dC3b^s}{dt}\right|_{max}}. \tag{S81}$$

From **Supplementary Equation S74** we determine the maximal flow of the $C3b^s$ concentration $\left|\frac{dC3b^s}{dt}\right|_{max}$. The results for varied fH^s concentrations are summarized in **Supplementary Figure S8A**. The flow drops monotonous with increasing fH^s concentration and has a significant drop starting right above fH^s_{th}. The maximal flow in the analyzed fH^s interval is about $10^{-5}\ \frac{\mu mol}{s \cdot dm^2}$. The time scale of the

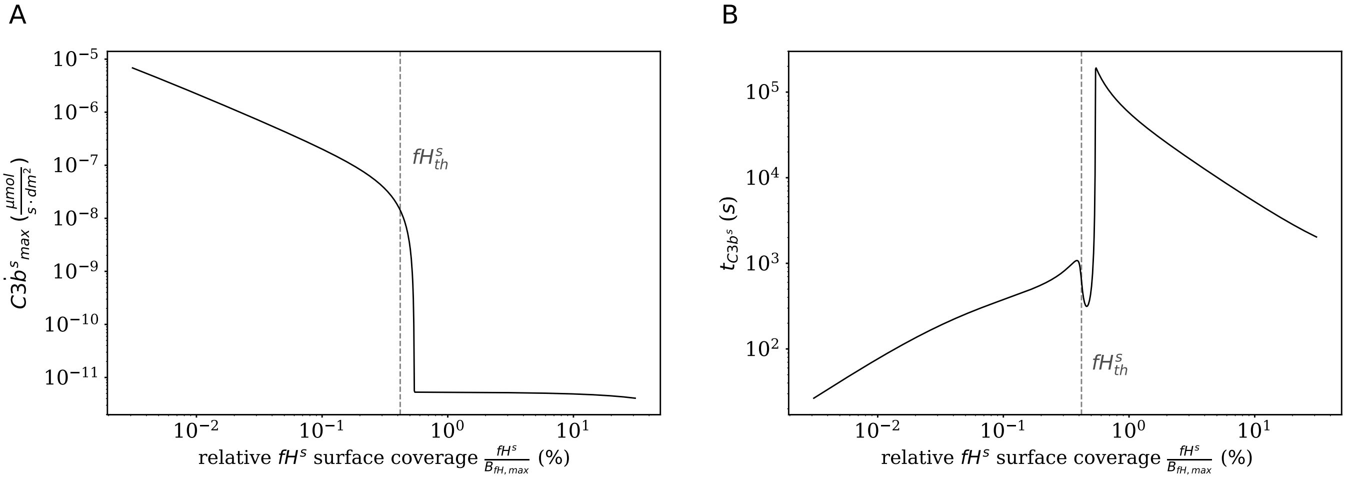

Figure S8. Analyzing maximal change and time scale of $C3b^s$ molecule concentrations for varying surface bound Factor H concentrations. **(A)** The maximal change of the $C3b^s$ molecule concentration is indirectly proportional to the fH^s concentration. **(B)** The time scale of the $C3b^s$ molecule concentration ranges from $3\,s$ to several hours.

$C3b^s$ concentration uses the steady state to determine the maximum change of the $C3b^s$ concentration and is shown in **Supplementary Figure S8B**.

We compare this time scale with the time scale of the QSSAs from **Supplementary Sections 1.4 - 1.6** and can conclude that the time scales $t_{C3b^f(R)}$ and t_{C3b^sH} are much smaller than t_{C3b^s} (see **Supplementary Figures S9A** and **S9B**). Thus, QSSAs for the $C3b^f(R)$ and $C3b^sH$ concentrations are justified. The time scales of $C3bBb$ and $C3bBbP$ (see **Supplementary Figure S9C**) are larger than the time scale of the $C3b^s$ concentration for fH^s concentrations below fH^s_{th}. Thus a QSSA for the given parameter set would cause large errors in the dynamics of the system. We decide to determine the dynamics of our model without a QSSA of the amplification process. The complete system, whose dynamics are calculated, is therefore:

$$\begin{aligned}\frac{d}{dt}C3b^s = & \; r_{ops} \cdot B_{C3b,free}(C3b^s, iC3b^s) \cdot C3b^f(R)^* - r^s_{inhib}(fH^s) \cdot C3b^s \\ & - r^+_{C3bB} \cdot fB^f \cdot C3b^s + r^-_{C3bB} \cdot C3b^sB \\ & + \left(r^-_{C3bBb} + r^-_{C3bBb,fH} \cdot fH^s\right) C3b^sBb \\ & + \left(r^-_{C3bBbP} + r^-_{C3bBbP,fH} \cdot fH^s\right) \cdot C3b^sBbP,\end{aligned} \tag{S82}$$

$$\frac{d}{dt}C3b^sB = r^+_{C3bB} \cdot fB^f \cdot C3b^s - \left(\frac{r_{C3bBb,cat} \cdot fD^f}{K_{cat,fD} + fD^f} + r^-_{C3bB}\right) \cdot C3b^sB, \tag{S83}$$

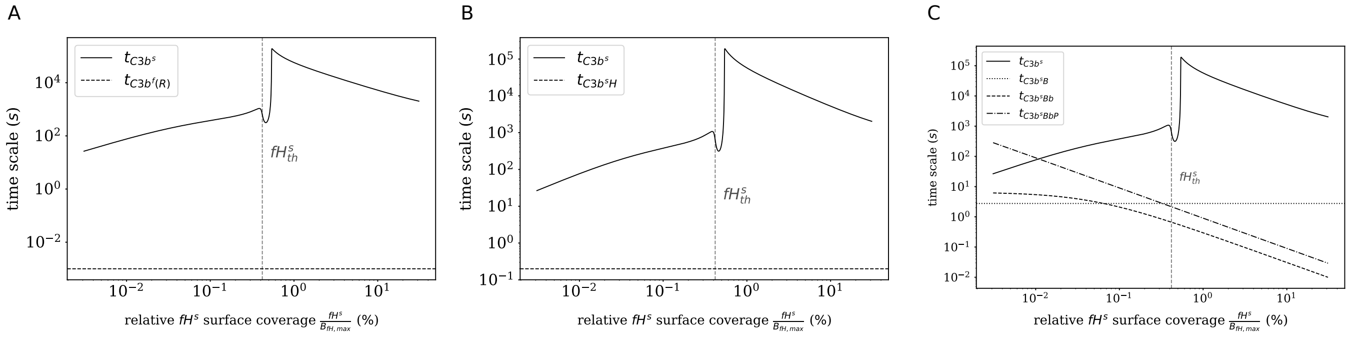

Figure S9. Analyzing time scales of the $C3b^s$ molecule concentrations and the QSSA time scales. **(A)** The time scale of the $C3b^f(R)$ concentration is at least four orders of magnitude smaller than the time scale of the $C3b^s$ concentration. **(B)** The time scale of the $C3b^sH$ concentration is at least two orders of magnitude smaller than the time scale of the $C3b^s$ concentration **(C)** For fH^s concentrations below fH^s_{th} the time scales of $C3bBb$ and $C3bBbP$ are larger than the time scale of the $C3b^s$ concentration. A QSSA would cause errors in the dynamics of the system.

$$\frac{d}{dt}C3b^sBb = \frac{r_{C3bBb,cat} \cdot fD^f}{K_{cat,fD} + fD^f} \cdot C3b^sB - \left(r^+_{C3bBbP,fP} \cdot fP^f + r^-_{C3bBb} + r^-_{C3bBb,fH} \cdot fH^s\right) \cdot C3b^sBb, \tag{S84}$$

$$\frac{d}{dt}C3b^sBbP = r^+_{C3bBbP,fP} \cdot fP^f \cdot C3b^sBb - \left(r^-_{C3bBbP} + r^-_{C3bBbP,fH} \cdot fH^s\right) \cdot C3b^sBbP, \tag{S85}$$

$$\frac{d}{dt}iC3b^s = r^s_{inhib}(fH^s) \cdot C3b^s - r_{dec} \cdot iC3b^s. \tag{S86}$$

We solve the dynamics using the fourth-order Runge-Kutta formula RK4 [45].

3.2 Quantitative prediction of Factor H mediated complement evasion

In order to analyze the influence of the parameters of the DynaCoSys model, we performed a local sensitivity analysis of the steady state of the model. With the local sensitivity analysis we pay attention to a particular point in the parameter space, our standard parameter set, defined by the paraemter values as given in the **Supplementary Tables** of **Supplementary Section 2.1**. The local sensitivity analysis will measure, how strong a change of the parameter changes the steady state. In the following we will shortly introduce the sensitivity analysis. A more detailed introduction can be found here [46].

A dynamical system $\vec{y}$ with set of parameters p is given by

$$\dot{\vec{y}}(t) = \vec{f}(\vec{y(t)}, p). \tag{S87}$$

The sensitivity of variable y_i with respect to parameter p is defined by $\frac{\partial y_i}{\partial p}$. If we consider only the steady state of the dynamical system, **Supplementary Equation S87** simplifies to

$$\vec{0} = \vec{f}(\vec{y(t)}, p). \tag{S88}$$

By differentiation with respect to the parameter

$$\vec{0} = \frac{\partial \vec{f}}{\partial \vec{y}} \frac{\partial \vec{y}}{\partial p} + \frac{\partial \vec{f}}{\partial p} \tag{S89}$$

and by solving for absolute local sensitivity we get

$$\frac{\partial \vec{y}}{\partial p} = -\left[\frac{\partial \vec{f}}{\partial \vec{y}}\right]^{-1} \frac{\partial \vec{f}}{\partial p}. \tag{S90}$$

with the following definitions

$$\left[\frac{\partial \vec{f}}{\partial \vec{y}}\right] = \begin{bmatrix} \frac{\partial f_1}{\partial y_1} & \frac{\partial f_1}{\partial y_2} & \cdots & \frac{\partial f_1}{\partial y_n} \\ \frac{\partial f_2}{\partial y_2} & \frac{\partial f_2}{\partial y_2} & \cdots & \frac{\partial f_2}{\partial y_n} \\ \cdots & \cdots & \cdots & \cdots \\ \frac{\partial f_n}{\partial y_1} & \frac{\partial f_n}{\partial y_2} & \cdots & \frac{\partial f_n}{\partial y_n} \end{bmatrix} \qquad \frac{\partial \vec{y}(t)}{\partial \vec{p}} = \begin{bmatrix} \frac{\partial y_1}{\partial p} \\ \frac{\partial y_2}{\partial p} \\ \cdots \\ \frac{\partial y_n}{\partial p} \end{bmatrix} \qquad \frac{\partial \vec{f}(t)}{\partial \vec{p}} = \begin{bmatrix} \frac{\partial f_1}{\partial p} \\ \frac{\partial f_2}{\partial p} \\ \cdots \\ \frac{\partial f_n}{\partial p} \end{bmatrix} \tag{S91}$$

where $\left[\frac{\partial \vec{f}}{\partial \vec{y}}\right]$ denotes the Jacobian matrix of the system. In order to compare the sensitivities of different parameters, we calculate the relative local sensitivities, that are computed as ratios of the relative changes

$$\frac{\frac{\partial \vec{y}}{y}}{\frac{\partial p}{p}} = \frac{p}{y} \frac{\partial \vec{y}}{\partial p}. \tag{S92}$$

The local sensitivities of the analyzed parameters share the common characteristic of highest absolute sensitivity at fH_{th}^s (see **Supplementary Figure S9**). The sensitivity of the parameters r_{amp}, r_{ops} and A

is in the complete analyzed Factor H interval positive, meaning an increased molecule concentration for increased parameter values. The influence of the parameters r^s_{inhib} and r_{dec} differs depending on molecule $C3b^s$ and $iC3b^s$.

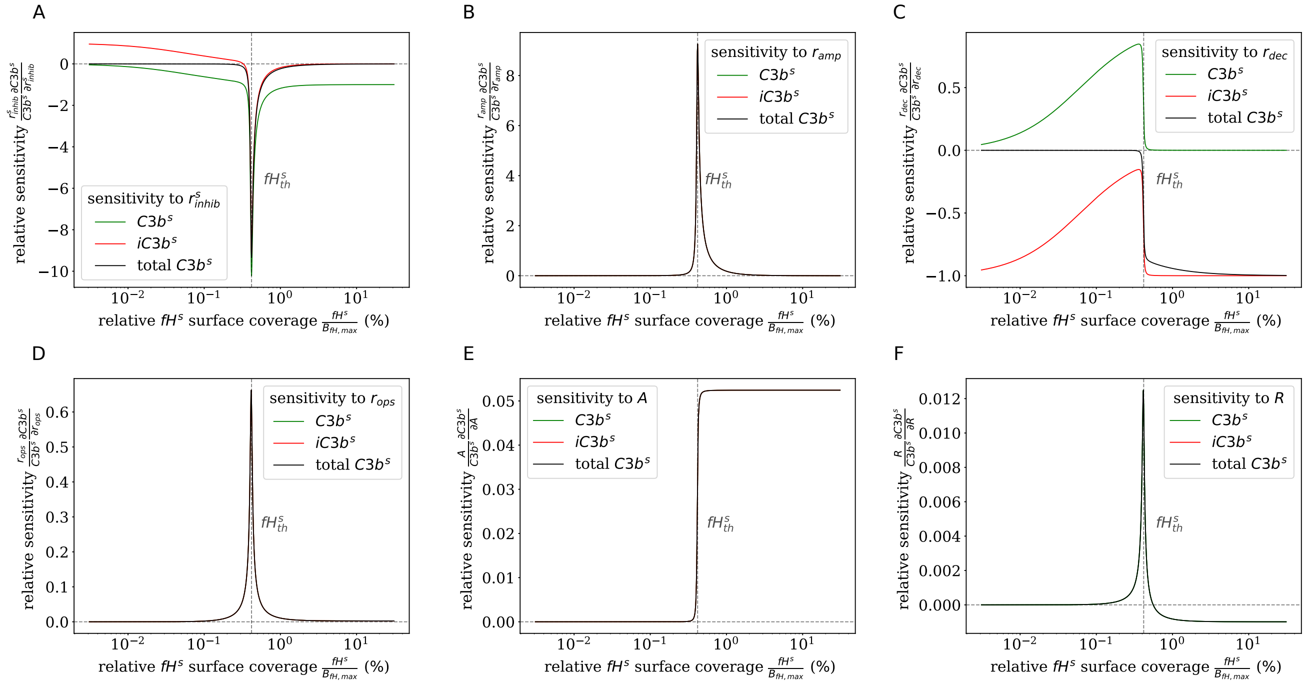

Figure S10. Local sensitivity analysis of the steady state of $C3b^s$, $iC3b^s$ and all surface bound $C3b$ molecules with respect to the parameters. **(A)** r^s_{inhib}, **(B)** r_{amp}, **(C)** r_{dec}, **(D)** r_{ops}, **(E)** A and **(F)** the cell radius R.

3.3 Complement model parameters have differential impact on critical Factor-H concentration

In this section, we investigate how the opsonization level, all C3b molecules on the surface, behaves in relation to the complement molecule concentration in the fluid. The most abundant complement molecule in the fluid is $C3^f$, which is given in **Supplementary Figure S11A**. An increase in $C3^f$ concentration does not lead to a strong change in the opsonization level. Since $C3^f$ is cleaved via Michaelis-Menten kinetics (see Supplementary **Equations S11** and **S54**), saturation occurs here. For decreasing $C3^f$ concentrations the boundary between non-self and self regime shifts to smaller fH^s binding site concentrations, *i.e.* less cells are targeted by the complement system. An increased concentration of fH^f (**Supplementary Figure S11B**) and fI^f (**Supplementary Figure S11C**), the complement regulators, shifts the threshold between non-self and self regime to smaller fH^s binding site concentrations. Thus, less cells are opsonized. Factor H has a much stronger effect than Factor I, since Factor H is involved in the inactivation of $C3b^s$ to $iC3b^s$ and inhibits the amplification of $C3b^f$. The molecules fB^f (**Supplementary Figure S11D**), fD^f (**Supplementary Figure S11E**) and fP^f (**Supplementary Figure S11F**) are involved in the amplification of $C3b^f$. With increasing concentration, the concentration of fH^s binding sites that is required to protect the surface increases. The molecule Factor B has the largest influence.

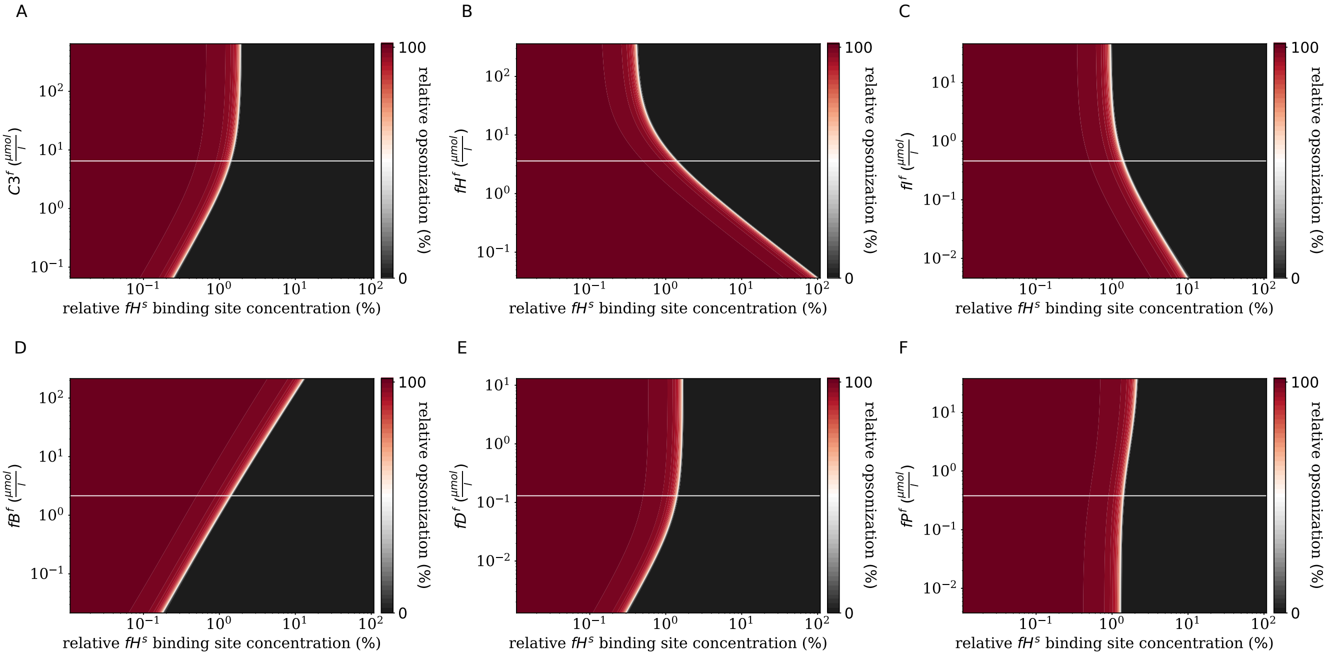

Figure S11. Steady state of relative opsonization for varied systems parameter and surface bound Factor H concentrations. **(A)** $C3^f$, **(B)** fH^f, **(C)** fI^f, **(D)** fB^f, **(E)** fD^f and **(F)** fP^f.

REFERENCES

[1]Peter F. Zipfel and Christine Skerka. Complement regulators and inhibitory proteins. *Nature Reviews Immunology*, 9(10):729–740, 2009.

[2]Marina Noris and Giuseppe Remuzzi. Overview of complement activation and regulation. *Seminars in Nephrology*, 33(6):479–492, 2013.

[3]Raffaella Parente, Simon J Clark, Antonio Inforzato, and Anthony J Day. Complement factor H in host defense and immune evasion. *Cellular and Molecular Life Sciences*, 74(9):1605–1624, 2017.

[4]Stephen J. Perkins and Mohammed Aslam. Four models of human factor H determined by solution scattering curve-fitting and homology modelling. *Protein Data Base*, 20.06.2019:https://www.rcsb.org/structure/1HAQ.

[5]M K Pangburn, R D Schreiber, and H J Müller-Eberhard. C3b deposition during activation of the alternative complement pathway and the effect of deposition on the activating surface. *The Journal of Immunology*, 131(4):1930–1935, 1983.

[6]Bert J C Janssen, Eric G. Huizinga, Hans C A Raaijmakers, Anja Roos, Mohamed R. Daha, Kristina Nilsson-Ekdahl, Bo Nilsson, and Piet Gros. Structures of complement component C3 provide insights into the function and evolution of immunity. *Nature*, 437(7058):505–511, 2005.

[7]Michael K. Pangburn and Hans J. Müller-Eberhard. The alternative pathway of complement. *Springer Seminars in Immunopathology*, 7(2-3):163–192, 1984.

[8]Michael K. Pangburn and Hans J. Müller-Eberhard. The C3 convertase of the alternative pathway of human complement. *Biochemical Journal*, 235:723–730, 1986.

[9]Timothy C Farries and Richard A Harrisont. the interactions between properdin, the third component. *Biochemical Journal*, 252:47–54, 1988.

[10]Jin Wu, You Qiang Wu, Daniel Ricklin, Bert J C Janssen, John D. Lambris, and Piet Gros. Structure of complement fragment C3b-factor H and implications for host protection by complement regulators. *Nature Immunology*, 10(7):728–733, 2009.

[11]M. K. Pangburn. Human complement C3b inactivator: isolation, characterization, and demonstration of an absolute requirement for the serum protein beta1H for cleavage of C3b and C4b in solution. *Journal of Experimental Medicine*, 146(1):257–270, 1977.

[12]Michael K. Pangburn and Hans J. Müller-Eberhard. Initiation of the Alternative Complement Pathway Due To Spontaneous Hydrolysis of the Thioester of C3. *Annals of the New York Academy of Sciences*, 421(1):291–298, 1983.

[13]Stephen J. Perkins, Ruodan Nan, Keying Li, Sanaullah Khan, and Ami Miller. Complement Factor H-ligand interactions: Self-association, multivalency and dissociation constants. *ImmunobiologyImmunobiology*, 217(2):281–297, 2012.

[14]Hirohumi Hirayama, Kiyono Yoshii, Hidetomo Ojima, Norikazu Kawai, Shintaro Gotoh, and Yuzo Fukuyama. Linear systems analysis of activating processes of complement system as a defense mechanism. *Biosystems*, 39(3):173–185, 1996.

[15]Andrey A Korotaevskiy, Leonid G Hanin, and Mikhail A Khanin. Non-linear dynamics of the complement system activation. *Mathematical Biosciences*, 222(2):127–143, 2009.

[16]Nehemiah Zewde, Ronald D Gorham, Angel Dorado, and Dimitrios Morikis. Quantitative Modeling of the Alternative Pathway of the Complement System. *PLOS ONE*, 11(3), 2016.

[17]Michael K. Pangburn, Robert D. Schreiber, and Müller-Eberhard Hans J. Formation of the initial C3 Convertase of the alternative complement pathway. *J. Exp. Med.*, 154:856 – 867, 1981.

[18]Ashok Reddy Dinasarapu, Anjana Chandrasekhar, Arvind Sahu, and Shankar Subramaniam. Complement C3. *Ucsd Molecule Pages*, 1(2):1–11, 2012.

[19]Michael K. Pangburn, Viviana P. Ferreira, and Claudio Cortes. Discrimination between host and pathogens by the complement system. *Vaccine*, 26(SUPPL. 8):15–21, 2008.

[20]Evelien T.M. Berends, Ronald D. Gorham, Maartje Ruyken, Jasper A. Soppe, Hatice Orhan, Piet C. Aerts, Carla J.C. de Haas, Piet Gros, and Suzan H.M. Rooijakkers. Molecular insights into the surface-specific arrangement of complement C5 convertase enzymes. *BMC Biology*, 13(1), 2015.

[21]Bert J.C. Janssen, Agni Christodoulidou, Andrew McCarthy, John D. Lambris, and Piet Gros. Human Complement Component C3b. *Protein Data Base*, 20.06.2019:http://www.rcsb.org/structure/2I07.

[22]Michael K. Pangburn and Hans J. Mueller-Eberhard. Kinetic and thermodynamic analysis of the control of C3b by the complement regulatory proteins factors H and I. *Biochemistry*, 22(1):178–185, 1983.

[23]Nehemiah Zewde Morikis and Dimitrios. A Computational Model for the Evaluation of Complement System Regulation under Homeostasis, Disease, and Drug Intervention. *PLOS ONE*, 2018.

[24]Lee A. Segel and Marshall Slemrod. The Quasi-Steady-State Assumption: A Case Study in Pertubation. *SIAM Review*, 31(3):446–477, 1989.

[25]Michael A. Kerr. Factor B and the Alternative Pathway C3/C5 Convertase. *Handbook of Proteolytic Enzymes*, 3:2869–2875, 2013.

[26]Dennis E Hourcade, Lynne M Mitchell, and M Edward Medof. Decay acceleration of the complement alternative pathway C3 convertase. *Immunopharmacology*, (42):167–173, 1999.

[27]Dennis V Pedersen, Lubka Roumenina, Rasmus K Jensen, Chiara Marinozzi, Capucine Picard, Tania Rybkine, Steffen Thiel, Cordula Stover, and Gregers R Andersen. Functional and structural insight into properdin control of complement alternative pathway amplification. *The EMBO Journal*, 36(8):1084–1099, 2017.

[28]D. T. Fearon. Properdin: binding to C3b and stabilization of the C3b-dependent C3 convertase. *Journal of Experimental Medicine*, 142(4):856–863, 1975.

[29]R G DiScipio. The binding of human complement proteins C5, factor B, beta 1H and properdin to complement fragment C3b on zymosan. *The Biochemical journal*, 199(3):485–496, 1981.

[30]Dennis E. Hourcade. The role of properdin in the assembly of the alternative pathway C3 convertases of complement. *Journal of Biological Chemistry*, 281(4):2128–2132, 2006.

[31]Arvind Sahu and Michael K. Pangburn. Covalent Attachment of Human Complement C3 to IgG. *The Journal of Biological Chemistry*, 269(46), 1994.

[32]Bing Liu, Jing Zhang, Pei Yi Tan, David Hsu, Anna M. Blom, Benjamin Leong, Sunil Sethi, Bow Ho, Jeak Ling Ding, and P. S. Thiagarajan. A computational and experimental study of the regulatory mechanisms of the complement system. *PLoS Computational Biology*, 7(1), 2011.

[33]Adithya Sagar, Wei Dai, Mason Minot, and Jeffrey D Varner. Reduced order modeling and analysis of the human complement system. *PLoS ONE*, 12(11), 2017.

[34]Volkmar Heinrich, Wooten D. Simpson, and Emmet A. Francis. Analytical Prediction of the Spatiotemporal Distribution of Chemoattractants around Their Source: Theory and Application to Complement-Mediated Chemotaxis. *Frontiers in Immunology*, 8:1–18, 2017.

[35]M Michael Glovsky, Peter A Ward, and Kent J Johnson. Complement determinations in human disease. *Annals of allergy, asthma & immunology : official publication of the American College of Allergy, Asthma, & Immunology*, 93(6):513–22, 2004.

[36]G Bajic, L Yatime, A Klos, and G.R. Andersen. Crystal Structure of the Human C3a anaphylatoxin. *Protein Data Base*, 20.06.2019:https://www.rcsb.org/structure/4HW5.

[37]DE Isenman and Cooper NR. The structure and function of the third component of human complement—I. The nature and extent of conformational changes accompanying C3 activation. *Molecular Immunology*, 18(4):331–339, 1981.

[38]Alfred H J Kim, Vibeke Strand, Deepali P Sen, Qiang Fu, Nancy L Mathis, Martin J Schmidt, Robin R Bruchas, Nick R Staten, Paul K Olson, Chad M Stiening, and John P Atkinson. Blood Concentrations of Complement Split Product iC3b and Serum C3 Associate with SLE Disease Activity. *Arthritis & Rheumatology*, 71, 2018.

[39]Michel D. Kazatchkine, Douglas T. Fearon, and K. Frank Austen. Human Alternative Complement Pathway: Membrane-Associated Sialic Acid Regulates the Competition between B and β1H for Cell-Bound C3b. *The Journal of Immunology*, 122(1):75–81, 1979.

[40]Mason X. Zhang and Bruce Klein. Activation, binding, and processing of complement component 3 (C3) by Blastomyces dermatitidis. *Infection and Immunity*, 65(5):1849–1855, 1997.

[41]Meike Heurich, Nigel J Francis, Dawn L Roberts, B Paul Morgan, and Claire L Harris. Common polymorphisms in C3, factor B, and factor H collaborate to determine systemic complement activity and disease risk. *Proc. Nati. Acad. Sci. USA*, 108(21):1–6, 2011.

[42]Claire L Harris, Rachel J M Abbott, Richard A Smith, and Susan M Lea. Molecular Dissection of Interactions between Components of the Alternative Pathway of Complement and Decay Accelerating Factor (CD55)*. *The Journal of Biological Chemistry*, 280(4):2569–2578, 2005.

[43]Frederick R Taylor, Sarah A Bixler, Joe I Budman, Dingyi Wen, Michael Karpusas, Sarah T Ryan, Gary J Jaworski, Abbas Safari-fard, Stuart Pollard, and Adrian Whitty. Induced Fit Activation Mechanism of the Exceptionally Specific Serine Protease, Complement Factor D †. *Biochemistry*, pages 2849–2859, 1999.

[44]Alex S. K. Law and Alister W. Dodds. The internal thioester and the covalent binding properties of the complement proteins C3 and C4. *Protein Science*, 6:263–274, 1997.

[45]William H. Press, Saul A. Teukolsky, William T. Vetterling, and Brian P. Flannery. Numerical recipes 3rd edition: The art of scientific computing. 2007.

[46]Brian Ingalls. Sensitivity analysis: from model parameters to system behaviour. *Essays in biochemistry*, 45:177–193, 2008.
